# Structural basis for small molecule targeting of Doublecortin Like Kinase 1 with DCLK1-IN-1

**DOI:** 10.1038/s42003-021-02631-y

**Published:** 2021-09-20

**Authors:** Onisha Patel, Michael J. Roy, Ashleigh Kropp, Joshua M. Hardy, Weiwen Dai, Isabelle S. Lucet

**Affiliations:** 1 The Walter and Eliza Hall Institute of Medical Research, Parkville, VIC Australia; 2 Department of Medical Biology, University of Melbourne, Parkville, VIC Australia

**Keywords:** Structural biology, X-ray crystallography

## Abstract

Doublecortin-like kinase 1 (DCLK1) is an understudied bi-functional kinase with a proven role in tumour growth and development. However, the presence of tissue-specific spliced DCLK1 isoforms with distinct biological functions have challenged the development of effective strategies to understand the role of DCLK1 in oncogenesis. Recently, DCLK1-IN-1 was reported as a highly selective DCLK1 inhibitor, a powerful tool to dissect DCLK1 biological functions. Here, we report the crystal structures of DCLK1 kinase domain in complex with DCLK1-IN-1 and its precursors. Combined, our data rationalises the structure-activity relationship that informed the development of DCLK1-IN-1 and provides the basis for the high selectivity of DCLK1-IN-1, with DCLK1-IN-1 inducing a drastic conformational change of the ATP binding site. We demonstrate that DCLK1-IN-1 binds DCLK1 long isoforms but does not prevent DCLK1’s Microtubule-Associated Protein (MAP) function. Together, our work provides an invaluable structural platform to further the design of isoform-specific DCLK1 modulators for therapeutic intervention.

## Abstract (web summary)

Patel et al. report crystal structures of the kinase domain of Doublecortin-like kinase 1 (DCLK1) in complex with a potent inhibitor and its precursors. Their structural study reveals insights into the mode of action of the inhibitor DCLK1-IN-1 and sets the basis for the design of isoform-specific inhibitors of DCLK1, which plays a role in tumour growth and development.

## Introduction

**Fig. 1:**
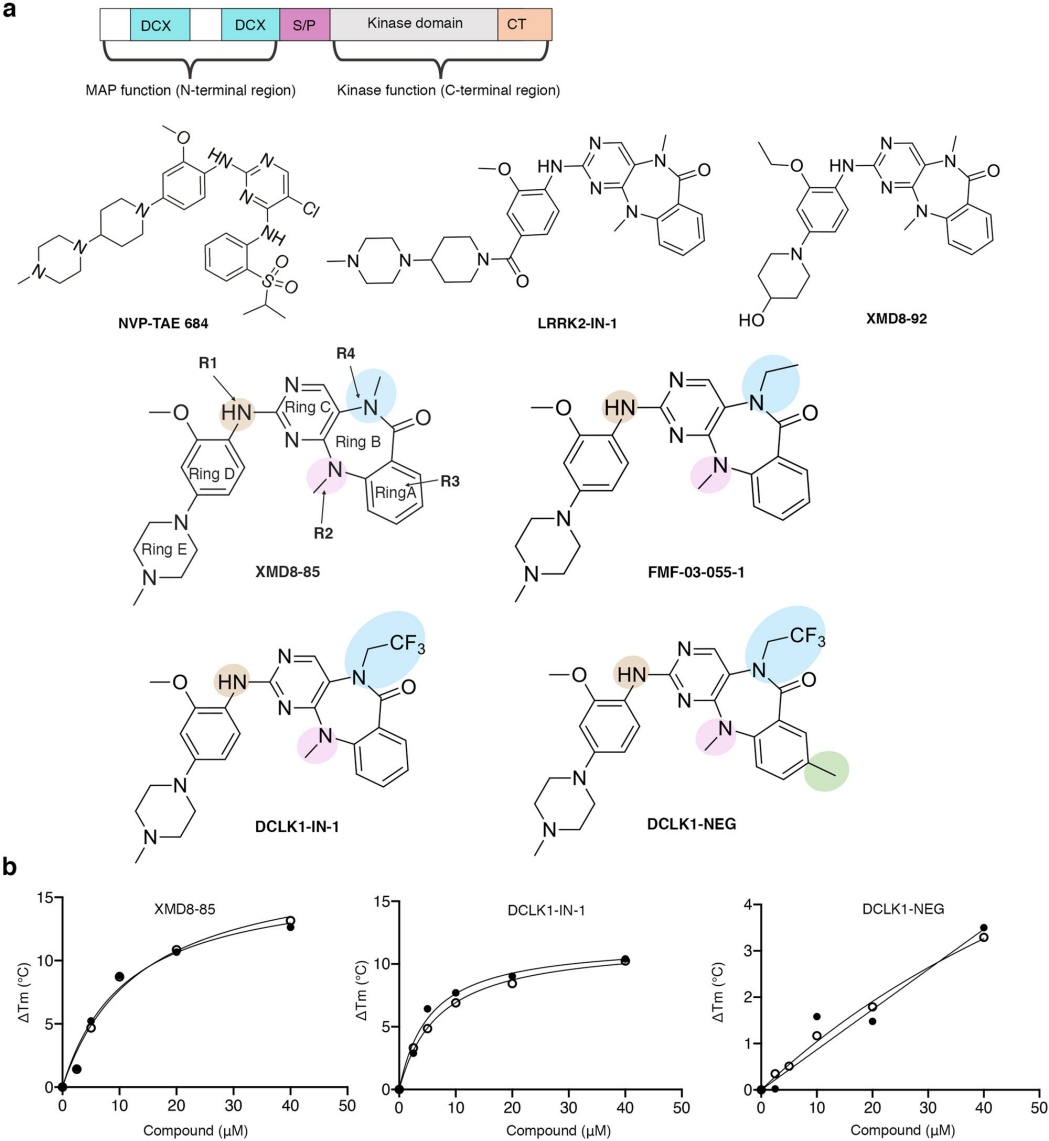
Benzopyrimido-diazipinone scaffolds and their binding to DCLK1-KD. **a** DCLK1 domain architecture highlighting the DCX domains at the N-terminal region and the kinase domain with its C-terminal regulatory tail (CT) at the C-terminal region. The N-terminal and the C-terminal regions are connected by the serine/proline (S/P) rich linker. Chemical structures of NVP-TAE684 and benzopyrimido-diazipinone scaffolds. R1, R2, R3, and R4 positions previously described^28^ are labelled and coloured. **b** Change in melting temperature (Δ*T*_m_) of DCLK1-KD  as a function of the concentration of XMD8-85, DCLK1-IN-1 and DCLK1-NEG. Change in the Δ*T*_m_ was calculated from the melting profiles shown in Supplementary Fig. 1. Each data point represents the calculated Δ*T*_m_ calculated for each inhibitor concentration; shown in duplicate experiments are shown.

Doublecortin-like kinase 1 (DCLK1) is a multi-domain bi-functional protein that belongs to the protein kinase superfamily and the doublecortin (DCX) family within the microtubule-associated protein (MAP) superfamily. The tandem doublecortin domain (DCX1 and DCX2), located in the N-terminal region of DCLK1, drives its MAP function, while the C-terminal region harbours a serine/threonine kinase domain (Fig. 1a).

Beyond its established role in neurogenesis, DCLK1 has been identified as an intestinal and pancreatic stem cell maker^1–3^ and growing evidence supports a role for DCLK1 in various malignancies. Many MAPs are known to play an important role in the regulation of microtubule dynamics and changes in their expression levels are often associated with the development and progression of cancer^4,5^. Overexpression of DCLK1 has been reported in multiple cancers, including colon, pancreatic, renal cell carcinoma and rectal neuroendocrine tumours^6–10^. Additionally, a relative high number of DCLK1 mutations have been identified in human gastric tumours^11,12^.

The regulation of DCLK1 is highly complex, and remains poorly understood. Multiple isoforms of DCLK1 exist, generated by alternative splicing^13–15^. These isoforms differ drastically in their domain composition and hence in their biological function. The most relevant functional differences between these isoforms lie in the presence or absence of the N-terminal tandem DCX domains that contribute to DCLK1’s MAP function. Two main human isoforms have been described, DCLK1-long (DCLK1-L), which contains the tandem DCX domains at the N-terminus and a kinase domain at the C-terminus and DCLK1-short (DCLK1-S), which contains only the C-terminal kinase domain^14,16,17^. For each of the DCLK1-L and -S isoforms, two other splicing variants exist (α and β), which differ in the length and sequence of the C-terminal regulatory tail that immediately follows the kinase domain. Unfortunately, the nomenclature used to describe the various DCLK1 isoforms has not been consistent over the years, which has led to some discrepancy^8,17^. In addition, commercial antibodies only specifically target a C-terminal sequence that is not present in all isoforms^3,9,17^. Hence, the overall expression profile of DCLK1 isoforms in various tissues, and their relative contribution to tumorigenesis have been largely overlooked. Adding to this complexity, recent studies have highlighted that DCLK1 undergoes epigenetic regulation. Hypermethylation of the DCLK1 5’(α) promoter in human colon adenocarcinomas (CRCs), results in the loss of the DCLK1-L isoform and the usage of an alternate β-promoter drives the expression of the DCLK1-S isoform^17–19^. In addition, the cleavage of DCLK1 by the cysteine protease calpain has been reported in neurons, resulting in the release of the kinase domain from the tandem doublecortin domains, a mechanism proposed to drive the relocalisation of the kinase domain to the nucleus^20^.

Despite the lack of mechanistic studies that pinpoint and dissect the expression pattern and the contribution of each DCLK1 isoform to tumorigenesis^21^, several studies have shown promising effects of DCLK1 knockdown or silencing on tumour growth in various cancer models^10,22–25^, highlighting DCLK1 as an attractive target. However, developing targeted strategies for such a protein with isoform-specific functions and varying expression levels is particularly challenging and is hampered by the lack of selective DCLK1 modulators that specifically target either DCLK1 kinase function or MAP function in isolation. Most studies reported to date have commonly used two DCLK1 kinase inhibitors based on a benzopyrimido−diazipinone scaffold, the LRRK2 compound, LRRK2-IN-1, and the ERK5 compound, XMD8-92, as they both showed off-target activity against DCLK1 kinase function^26,27^. However, their pan-selectivity makes the published studies difficult to interpret with respect to DCLK1 kinase function. Recently, the Gray group have generated a bespoke highly selective DCLK1/DCLK2 inhibitor (DCLK1-IN-1) derived from this benzopyrimido-diazipinone series, as a way forward for dissecting the contribution of DCLK1 kinase activity on DCLK1 tumorigenesis activity^28^.

Here, we present the crystal structures of the DCLK1 kinase domain in complex with DCLK1-IN-1 and these two critical intermediates. Our structural data rationalises the structure-activity relationship (SAR) behind DCLK1-IN-1 and aids in understanding the increased selectivity of DCLK1-IN-1 towards DCLK1 relative to ERK5 and LRRK2 kinases. Interestingly, the binding of DCLK1-IN-1 induces an opening of the ATP binding site, highlighting the plasticity of this site, a feature not observed in the other DCLK1 crystal structures solved to date. In addition, we rigorously investigate the complexity of DCLK1 regulation and demonstrate that whilst DCLK1-IN-1 binds to the DCLK1-long isoform with high affinity, it does not inhibit DCLK1 MAP function. Taken together, our structural data provide a framework for the generation of highly selective and highly potent DCLK1 inhibitors that target DCLK1 short isoforms. In addition, our work also provides the structural framework to further the design of chemical probes to enable a targeted DCLK1 degradation strategy, the approach most suited for therapeutically targeting proteins with multiple functions.

## Results

### DCLK1  binding to benzopyrimido−diazipinone scaffold molecules

Multiple compounds based on a 5, 11-dihydro-6H-benzo[e]pyrimido[5,4-b][1,4]diazepin-6-one scaffold (Fig. 1a) are known to have potent activity within the kinase family^29–32^. Within this compound class, the LRKK2 kinase inhibitor, LRRK2-IN^33^, and the ERK5 inhibitors XMD8-92 and XMD8-85^34^ displayed off-target activities against DCLK1 and DCLK2^34–36^ and so good starting point for further development as DCLK1 kinase inhibitors. Derived from this scaffold, DCLK1-IN-1 was recently reported as a selective DCLK1/DCLK2 ATP competitive inhibitor^28,37^. In particular, this was achieved through modifications at R1 and R4 positions, which enhanced DCLK1 selectivity of DCLK1-IN-1 by reducing binding to ERK5 and BRD4^28^ (Fig. 1a). Taking advantage of the availability of this potent series of DCLK1 benzopyrimido-diazipinone scaffold inhibitors, we were interested in undertaking crystallography studies in order to provide a more detailed structural framework to further guide the development of DCLK1 selective tools.

We first confirmed the ability of DCLK1-IN-1’s precursor, XMD8-85, and subsequently DCLK1-IN-1, to bind to the DCLK1 kinase domain (DCLK1-KD; residues 372-649) by carrying out thermal stability assays (Fig. 1b and Supplementary Fig. 1). We used as a structurally related negative control compound, DCLK1-NEG, reported to have reduced affinity for DCLK1, owing to the incorporation of an additional methyl substituent at R3 (Fig. 1a) that would be expected to cause a steric clash with residues located at the start of the activation loop and near the floor of the ATP binding site^21^. By performing a compound titration between 2.5 and 40 μM, we demonstrated an increased shift in the melting temperature of DCLK1-KD (ΔT_m_) as the concentration of the compounds increased, confirming that both XMD8-85 and DCLK1-IN-1 bind DCLK1-KD. As expected, DCLK1-NEG showed severely reduced binding to DCLK1-KD (Fig. 1b and Supplementary Fig. 1).

### Crystal structure of DCLK1-KD:XMD8-85

**Fig. 2:**
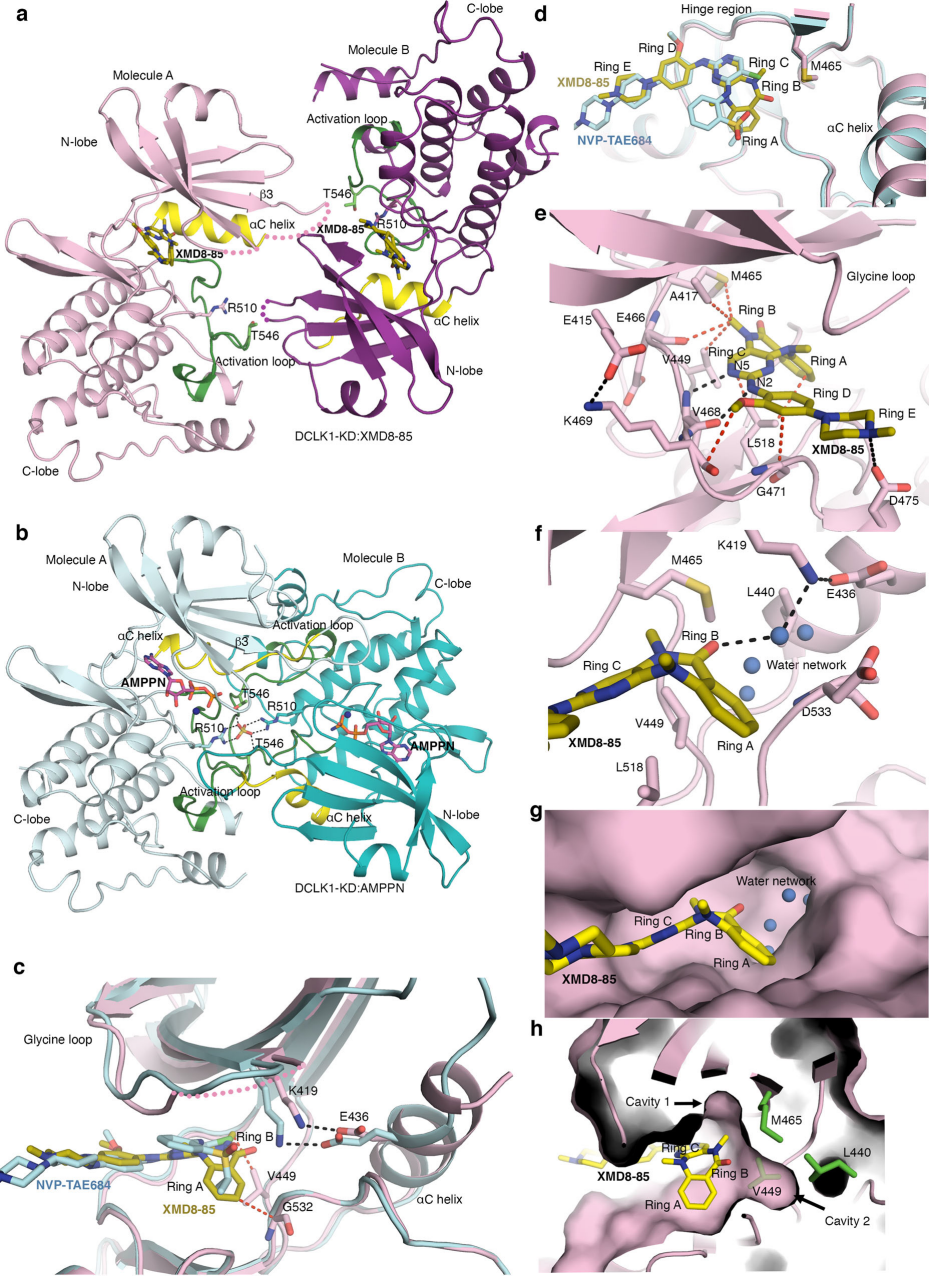
Structure of DCLK1-KD:XMD8-85. **a** The structure of two molecules of DCLK1-KD:XMD8-85 within the asymmetric unit in a “head-to-tail” arrangement. **b** The structure of two molecules of DCLK1-KD:AMPPN (PDB 5JZJ) within the asymmetric unit in a “head-to-tail” and “face-to-face” arrangement. **c** Overlay of DCLK1-KD:XMD8-85 with DCLK1-KD:NVP-TAE684 showing the conservation of the canonical salt-bridge between Glu436 and Lys419 and differences in the αC helix conformation. **d** Top view of the overlay of DCLK1-KD:XMD8-85 with DCLK1-KD:NVP-TAE684. XMD8-85, rings B and A sits deeper into the back pocket within the ATP binding site. **e** Close up of the interaction of DCLK1-KD with XMD-8-85. **f** Water mediated network formed by the diazepine ring B with the invariant Lys419 and Glu436. **g** Surface representation of DCLK1-KD:XMD8-85 to highlight the cavity around the water-mediated network described in **f**. **h** The DCLK1-KD:XMD8-85 crystal structure identified two cavities, cavity 1 and 2) near the back pocket within the ATP binding site that could be exploited to achieve selectivity towards DCLK1. The h-bond and van der Waals interaction are shown in black and red dashed lines, respectively. The missing residues in DCLK1-KD:XMD8-85 are shown in pink dashed lines. Water molecules are shown as blue spheres. AMPN, magenta; XMD8-85, yellow; NVP-TAE684, cyan.

To understand the structural basis by which XMD8-85 increased inhibition of DCLK1 and to elucidate its retained pan-activity against ERK5 and LRRK2, we solved the structure of DCLK1-KD (residues 372-649) in complex with XMD8-85 to 2.5 Å (Fig. 2a, Table 1, and Supplementary Fig. 2). The DCLK1-KD:XMD8-85 complex crystallised with two molecules in the asymmetric unit with a head to tail packing similar to DCLK1-KD:AMPPN (PDB 5JZJ) and DCLK1-KD:NVP-TAE684 (PDB 5JZN) crystal structures (Fig. 2a, 2b) that we described previously^21^. However, the “face-to-face” arrangement previously seen in these structures, promoted by the interaction of the extended αC helix from one molecule against the activation loop of the second molecule, is not observed in this new structure, likely due to an altered crystal packing. In the DCLK1-KD:AMPPN and DCLK1-KD:NVP-TAE684, this unusual activation loop dimerisation mode was found to be stabilised by the coordination of a sulfate molecule between Arg510 from the catalytic loop of one molecule and Thr546 from the activation loop of the other (Fig. 2b). In DCLK1-KD:XMD8-85, the sulfate coordination is no longer maintained. Despite this, the activation loop is visible in an open conformation with the first three conserved activation loop residues Asp-Phe-Gly (DFG) adopting a classical DFG-in conformation (Fig. 2a and Supplementary Fig. 3). In this conformation, the DFG phenylalanine packs into an hydrophobic grove and contribute to the intact regulatory and catalytic spines, indicative of an active conformation (Supplementary Fig. 4)^38^. This active conformation is further confirmed by the presence of the canonical salt-bridge interaction between the conserved αC helix glutamate (Glu436) and the invariant β3 strand lysine (Lys419) (Fig. 2c). However, the extended αC helix conformation previously reported is not seen (Fig. 2a, b)^21^. Instead, an additional turn in the αC helix is visible (Fig. 2c). While the position of ring C of XMD8-85 aligns with NVP-TAE684, both rings A and B of XMD8-85 sit deeper into the back pocket within the ATP binding site (Fig. 2d). XMD8-85 is further stabilised by  vdw interactions between ring A and Gly532 (that precedes the DFG motif) and between ring B and Val449 (that is located between αC helix and β5 strand) (Fig. 2c). Mutation of Gly532 to valine considerably reduces XMD8-85 binding affinity for DCLK1-KD (Supplementary Fig. 5), likely by causing a steric clash.

**Table 1:** Data collection and refinement statistics (molecular replacement).

	DCLK1-IN-1	XMD8-85	FMF-03-055-1
*Data collection*			
Space group	C2	I2	P2_1_
Cell dimensions			
*a*, *b*, *c* (Å)	143.95, 61.71, 65.31	66.18, 63.33, 152.49	65.92, 62.43, 72.08
*α*, *β*, *γ* (°)	90.00, 103.04, 90.00	90.00, 100.47, 90.00	90.00, 96.07, 90.00
Resolution (Å)	44.29−3.00 (3.18−3.00)*	74.97−2.50 (2.60−2.50)	71.7−3.10 (3.31−3.10)
*R*_merge_	0.156 (0.775)	0.117 (0.674)	0.236 (0.927)
*I* / σ*I*	11.4 (3.6)	10.8 (2.9)	7.3 (2.6)
Completeness (%)	98.8 (93.0)	99.1 (99.7)	99.4 (99.8)
Redundancy	6.8 (6.8)	4.7 (4.8)	4.7 (4.8)
*Refinement*			
Resolution (Å)	44.29−3.00	74.97−2.50	71.7−3.10
No. reflections	76277 (11337)	101629 (11767)	50639 (9282)
*R*_work_/*R*_free_	0.2030/0.2597	0.1940/0.2251	0.1960/0.2508
No. atoms			
Protein	3935	3919	3943
Ligand/ion	96	68	70
Water	4	92	-
*B*-factors			
Protein	56.6	36.9	37.8
Ligand/ion	60.7	36.9	42.8
Water	39.8	30.7	-
R.m.s. deviations			
Bond lengths (Å)	0.007	0.004	0.006
Bond angles (°)	0.967	0.707	0.658

One crystal was used for each structure. *Values in parentheses are for highest-resolution shell.

The interaction of XMD8-85 with DCLK1-KD results in two hydrogen bonds interactions within the ATP binding site (Fig. 2e). These all occur through the interaction of XMD8-85 within the hinge region, involving a donor/acceptor hydrogen bond pair with the backbone of Val468. The secondary amine (N2) in the linker between rings D and C of XMD8-85 acts as a hydrogen bond donor for the backbone carbonyl oxygen of Val468, and the  pyrimidine nitrogen atom (N5) in ring C of XMD8-85 acts as an acceptor for the backbone amide -NH of Val468. Additionally, the hinge region residues Gly471 and Lys469 further stabilise ring D through main chain interactions. Residue Leu518, located on β7 at the floor of the ATP binding site, also stabilises rings A, B, and C through hydrophobic/vdw interactions. Notably, the amide carbonyl oxygen of the diazepine ring B participates in a network of water-mediated hydrogen bond interaction with the invariant Lys419 and αC Glu436 that play a critical role in nucleotide binding (Fig. 2f, g). The N-methyl amide substituent at position R4 (Fig. 1) on the diazepine ring B confers affinity for DCLK1. This R4 N-methyl group  appears to form favourable vdw interactions with the gatekeeper residue Met465 (on β5) located deep in the ATP binding site, as well as Ala417, Val449, and Glu466 (Fig. 2e). Removal of the N-methyl group has been demonstrated to substantially reduce affinity towards DCLK1, ERK5 or BRD4^28^.

Interestingly, the salt bridge interaction between residue Glu415 (β3 strand) and Lys469 from the hinge region is maintained, acting as an anchor point to maintain the structural integrity of this interface (Fig. 2e). The structure also revealed other features of XMD8-85 that likely account for its improved DCLK1 affinity relative to XMD8-92. Firstly, the ortho methoxy substituent in ring D in XMD8-85 is likely better accommodated in DCLK1 than the bulkier ortho ethoxy substituent of XMD8-92 as this bulkier substituent would be expected to cause unfavourable contacts with E406 of DCLK1; secondly, protonation of the N-methylpiperazine of XMD8-85 at physiological pH would enable an additional favourable electrostatic interaction with Asp475 of DCLK1, which is not possible for the 4-hydroxypiperidine substituent of XMD8-92^28^. However, the DCLK1-KD:XMD8-85 crystal structure also revealed aspects of sub-optimal shape complementarity of XMD8-85 for the DCLK1 ATP binding site and two additional cavities (cavity 1 and 2) near the back pocket that might be exploited to improve selectivity towards DCLK1 (Fig. 2g, h). Overall, our DCLK1-KD:XMD8-85 crystal structure clearly indicated that the N-methyl group in the R4 position is orientated towards the gatekeeper residue Met 465, such that modifications in this position might be used to improve specific selectivity for DCLK1, in particular, relative to other kinases with a differing gatekeeper residue.

### R4 modification provides increased selectivity towards DCLK1

Based on our initial structural information, two compounds were designed by Ferguson et al., introducing ethyl (FMF-03-055-1) and trifluoroethyl (DCLK1-IN-1) substituents at the R4 position (Fig. 1a)^28^ to improve selectivity towards DCLK1 by targeting the deep hydrophobic back pocket surrounded by residues Leu440, Val449, Met465 and the backbone of the DFG loop (Fig. 2h).

**Fig. 3:**
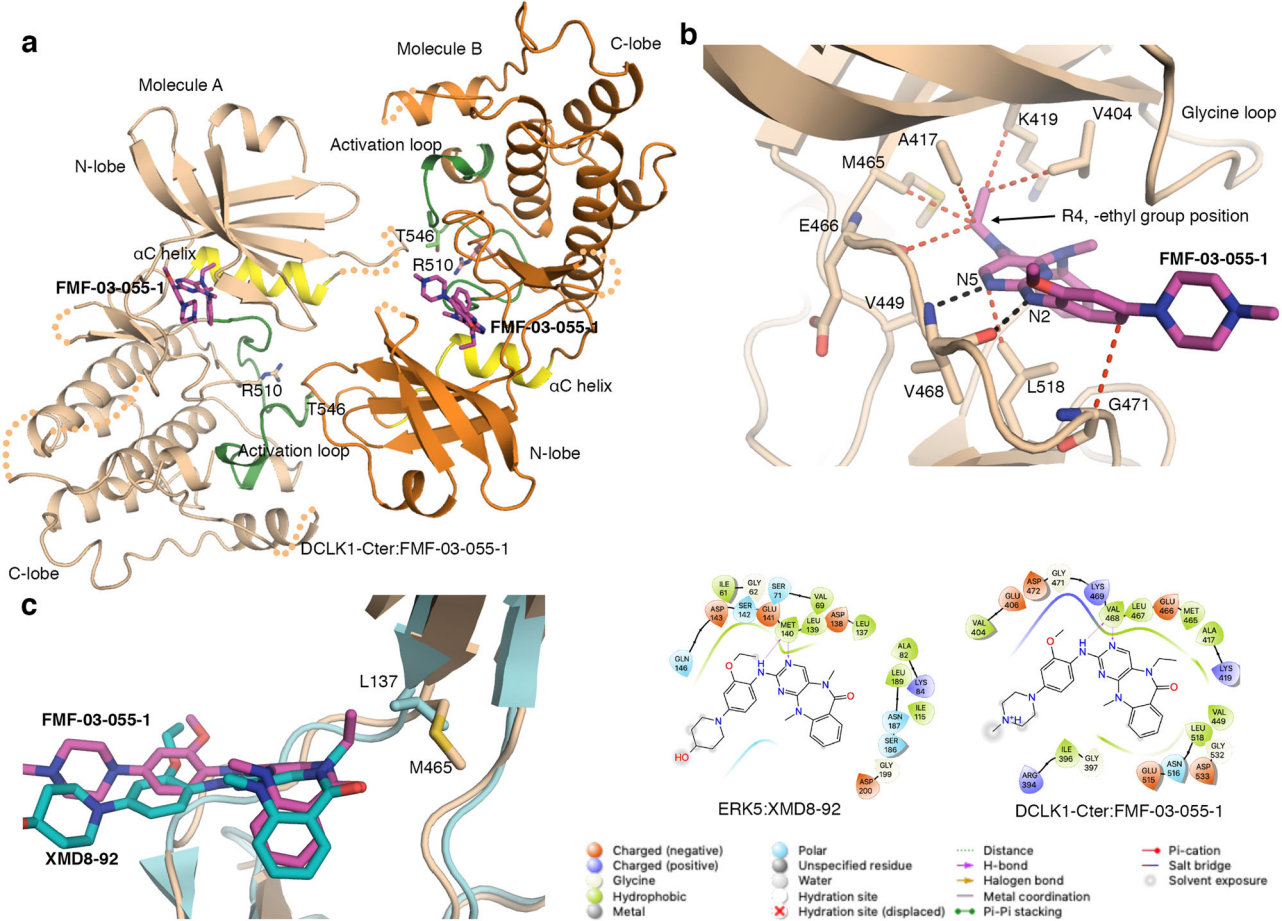
Structure of DCLK1-Cter:FMF-03-055-1. **a** The structure of two molecules of DCLK1-Cter:FMF-03-055-1 within the asymmetric unit in a “head-to-tail” arrangement. **b** Close up of the interaction of DCLK1-Cter with FMF-03-055-1. **c** Overlay of DCLK1-Cter:FMF-03-055-1with ERK5:XMD8-92 showing the differences in the gatekeeper residues, Leu137 in ERK5 and Met 465 in DCLK1 and ligand interaction diagrams generated using Schrödinger Maestro, including prediction of protonation states at pH 7.0 using Epik (Release 2020-3:, Schrödinger, LLC, New York, NY, 2020). The h-bond and vdw interaction are shown in black and red dashed lines, respectively. The flexible residues in DCLK1-Cter: FMF-03-055-1 are shown in orange dashed lines. FMF-03-055-1, magenta; XMD8-92, teal.

The introduction of an N-ethyl substitution at position R4 was shown to be well tolerated by DCLK1, resulting in 5-fold enhanced potency, as compared to XMD8-85^28,37^. This modification also reduced off-target binding to both ERK5 and BRD4, but only to a limited extent for LRRK2^28^. LRRK2 has identical residues to DCLK1 within the ATP binding site, including the gatekeeper methionine, which could explain this observed retention of potency (PDB 6VNO^39^). To better understand how the ethyl substitution at position R4 provided increased potency towards DCLK1 and increased selectivity over ERK5, we solved the crystal structure of DCLK1-Cter:FMF-03-055-1 (residues 372-686) to 3.1 Å resolution (Fig. 3a, Table 1, and Supplementary Fig. 2). The backbone H-bond interactions mediated by Val468 located in the hinge region are still conserved in DCLK1-Cter:FMF-03-055-1 (Fig. 3b). Our crystal structure reveals that the R4 ethyl group in FMF-03-055-1 indeed mediates additional contacts with Lys419, Ala417, Val404 and the gatekeeper residue Met465 as compared to the R4 methyl in XMD8-85 (Fig. 3b). In addition to enhancing binding to DCLK1, the R4 ethyl substitution also decreased off-target binding to ERK5^28^. This could be attributed to the presence in ERK5 of a leucine instead of methionine as the gatekeeper residue (Leu137). While a leucine residue is shorter, it has more constrained side-chain conformations than methionine. Analysis of the ERK5:XMD8-92 (PDB 5BYY) structure suggests that an ethyl group at position R4 would result in a clash with Leu137 in its current rotamer conformation (Fig. 3c). Our structural data, therefore, provides the rationale for the increased affinity of FMF-03-055-1 towards DCLK1 and its loss of affinity towards ERK5.

### DCLK1-IN-1 selectively binds DCLK1-KD

**Fig. 4:**
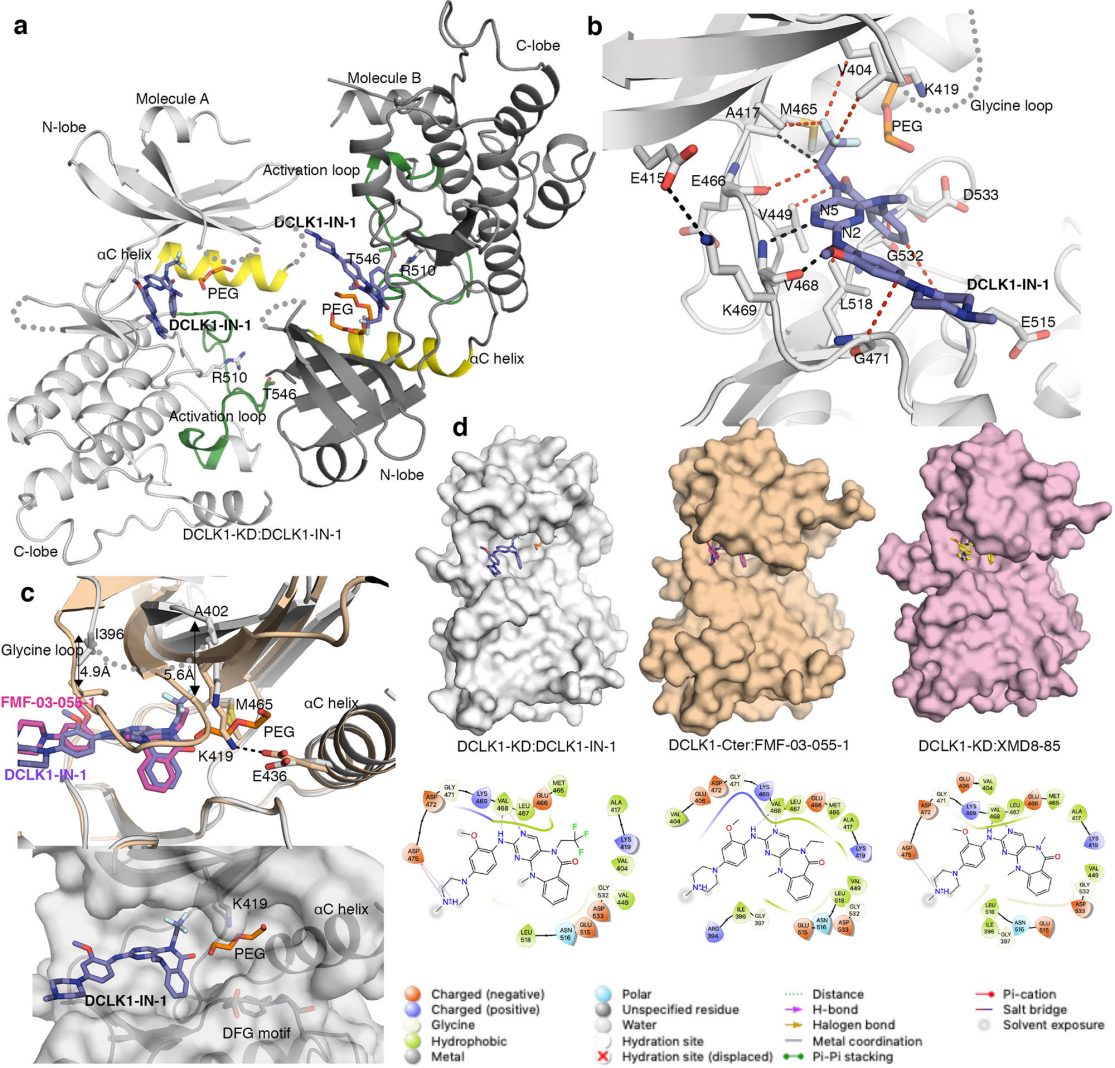
Structure of DCLK1-KD:DCLK1-IN-1. **a** The structure of two molecules of DCLK1-KD:DCLK1-IN-1 within the asymmetric unit in a “head-to-tail” arrangement. **b** Close up of the interaction of DCLK1-KD with DCLK1-IN-1. **c** Overlay of DCLK1-Cter:FMF-03-055-1 with DCLK1-KD:DCLK1-IN-1 to highlight the opening of the ATP binding site and surface representation of DCLK1-KD:DCLK1-IN-1 showing the site occupied by a PEG molecule. The region near the glycine loop undergoes an upward shift (arrows indicated) by 5 Å (distance measured at the Cα atom of Ile396 located at the start of the glycine loop and Ala402 located at the end of the loop) to accommodate the bulky trifluoroethyl group. **d** Surface representation of DCLK1-KD:XMD8-85, DCLK1-Cter:FMF-03-055-1 and DCLK1-KD:DCLK1-IN-1 and ligand interaction diagrams generated using Schrödinger Maestro. The missing residues in DCLK1-KD: DCLK1-IN-1 are shown in grey dashed lines. The h-bond and van der Waals interaction are shown in black and red dashed lines, respectively. DCLK1-IN-1, purple; FMF-03-055-1, magenta. See also Supplementary Figs. 6 and  7.

While the R4 ethyl group in FMF-03-055-1 enhanced affinity towards DCLK1 and decreased off-target affinity for ERK5, FMF-03-055-1 still showed appreciable binding to LRRK2 and BRD4^28^. To further probe the R4 position, an electronegative trifluoroethyl group was introduced instead of hydrophobic ethyl, which led to DCLK1-IN-1^37^. Interestingly, DCLK1-IN-1 showed a modest reduction in affinity for DCLK1 compared to FMF-03-055-1 but a simultaneous improvement in selectivity against ERK5, LRRK2, and BRD4^28^. To better understand the effect of this change, we crystallised DCLK1-KD in complex with DCLK1-IN-1 to 3.1 Å resolution (Fig. 4a, b and Supplementary Fig. 2). The DCLK1-KD:DCLK1-IN-1 complex crystallised in a similar head to tail conformation as seen for the DCLK1-KD:XMD8-85 and DCLK1-Cter:FMF-03-055-1 (Figs. 2a, 3a, and 4a). Overall, the substitution of the methyl or ethyl group at position R4 with the trifluoroethyl group does not affect the conformation of the Met465 side-chain nor the conformation of the αC helix, the A-loop, the catalytic loop, the hinge region (Fig. 4c and Supplementary Fig. 6) and the alignment of the regulatory and catalytic spines (Supplementary Fig. 4). However, a striking feature of the DCLK1-KD:DCLK1-IN-1 structure, is an opening of the ATP binding site to accommodate the bulkier trifluoroethyl group. The specific orientation adopted by the trifluoroethyl group allows additional contacts with residues Val404, Ala417 and Met465 (Fig. 4b). In addition, the N-lobe undergoes a twist that shifts the positions of the β strands between 2.5 and 3.5 Å. The region near the glycine loop also undergoes an upward shift by 5 Å (distance measured at the position of Cα of Ile396 located at the start of the glycine loop and Ala402 located at the end of the loop) (Fig. 4c, d and Supplementary Fig. 6b). Consequently, no electron density was observed for glycine loop residues 397−401, due to its flexibility. Notably, the presence of the trifluoroethyl group disrupts the salt-bridge interaction between the β3 strand invariant lysine (Lys419) and the conserved the αC helix glutamate (Glu436) (Fig. 4c). Interestingly, this opening creates a shallow pocket, which in our structure is occupied by an additional electron density seen in both copies of DCLK1 that could be attributed to a fragment of polyethylene glycol (PEG), a component of the crystallisation condition (Fig. 4c and Supplementary Fig. 2). Interestingly, the position of the PEG is in a similar location to an allosteric inhibitor bound to ERK5 (Supplementary Fig. 7)^34^.

Together, our structural data demonstrates that in contrast to XMD8-85 and FMF-03-055-1, which adopt a conventional type I binding mode (i.e., binding an active conformation of the DCLK1 kinase) (Figs. 2c and 3b), DCLK1-IN-1 adopts a binding mode akin to a type 1.5 binding mode, whereby the DFG-in conformation is maintained but the canonical salt bridge between the β3 strand lysine (Lys419) and the αC helix glutamate (Glu436) is disrupted (Fig. 4c). However, in contrast to other type 1.5 inhibitors, like lapatinib (bound to EGFRK, PDB 1XKK) or vemurafenib (bound to B-Raf, PDB 3OGY) the position of the αC helix glutamate is unchanged (Supplementary Fig. 7c). Conventional type 1.5 inhibitors are known to induce an αC helix-out conformation (conserved glutamate pointing out) by binding deeper into the ATP binding site, underneath the αC helix. In contrast, DCLK1-IN-1 binding directly causes the disruption of the canonical salt bridge by opening the ATP binding site, and leaving an unoccupied space underneath the αC. Overall, these changes can account for the dramatically increased DCLK1-IN-1 selectivity for DCLK1 against ERK5, LRRK2 and BRD4^28^ (Supplementary Table 1) and raise the possibility that the space occupied by the PEG molecule could be further explored for the design of DCLK1 selective inhibitors.

### DCLK1-IN-1 binds DCLK1-L isoforms

**Fig. 5:**
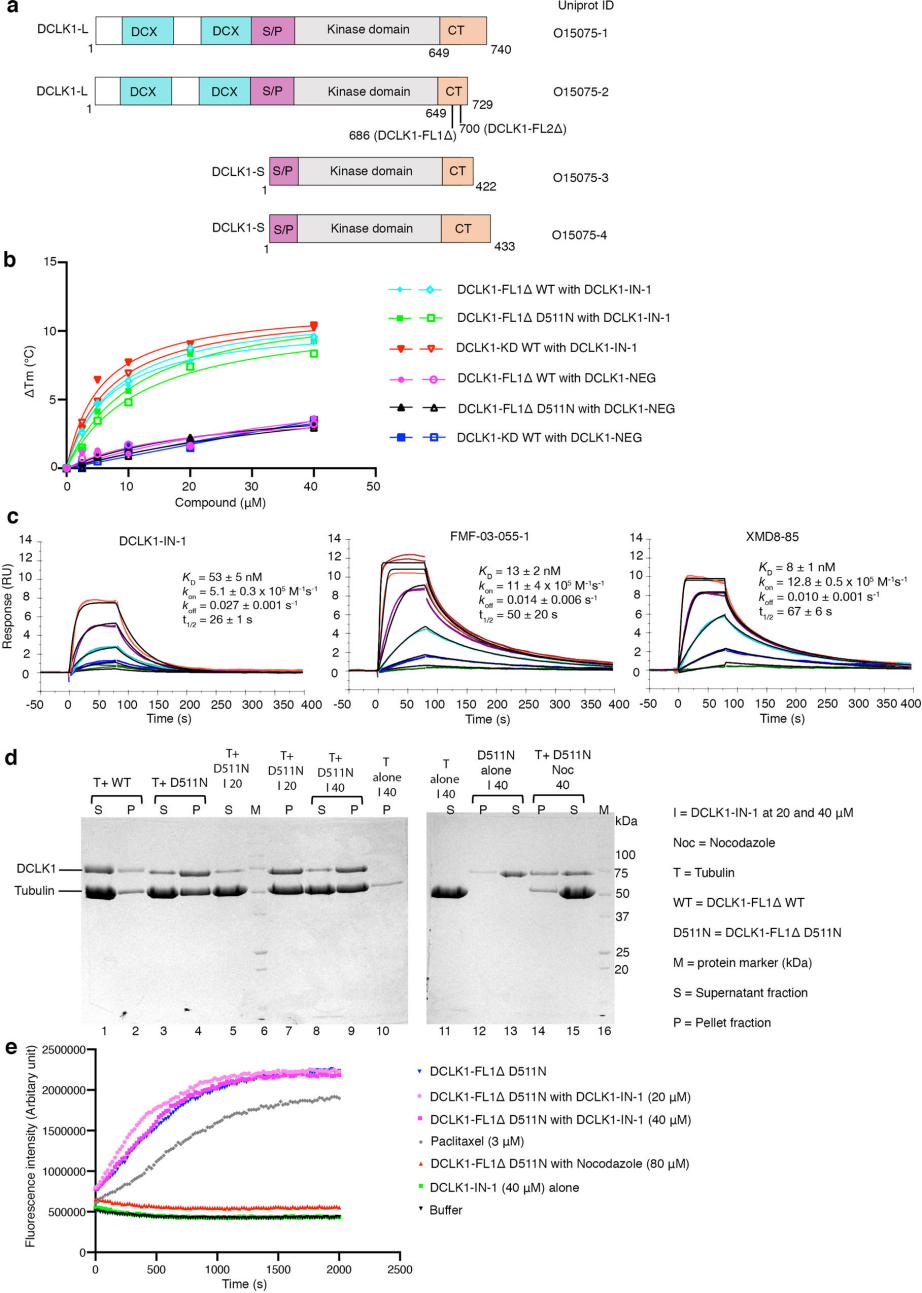
DCLK1 isoforms and the effect of DCLK1-IN-1 on microtubule polymerisation and binding function. **a** Domain diagrams of DCLK1 isoforms based on the Uniprot classification. **b** The difference in melting temperature (*T*_m_) of DCLK1-KD, DCLK1-FL1Δ D511N and DCLK1-FL1Δ WT in the presence of DCLK1-IN-1 and DCLK1-NEG. The difference in the *T*_m_ was calculated from the melting profiles shown in Supplementary Fig. 8. **c** Representative fitted SPR sensorgrams for DCLK1-IN-1, FMF-03-055-1, or XMD8-85 binding to immobilised DCLK1-FL1Δ D511N, showing mean values from kinetic fitting to a 1:1 binding model (*K*_D_, dissociation constant; *k*_on_, on-rate; *k*_off_, off-rate; *t*_1/2_, dissociative half-life for the protein/inhibitor complex). Data represent an average of either four (DCLK1-IN-1) or three (FMF-03-055-1 and XMD8-85) independent experiments, each performed as duplicate titrations; errors are SEM. See also Supplementary Fig. 9. **d** Microtubule polymerisation and pelleting assays followed by SDS-PAGE gel analysis of pellet (P) and supernatant (S) fractions. Tubulin polymerisation was tested in the presence of DCLK1-FL1Δ D511N and DCLK1-FL1Δ WT. The microtuble destabiliser, nocodazole, was used as a negative control. DCLK1-FL1Δ D511N was incubated with DCLK1-IN-1 (20 and 40 μΜ) or nocodazole (40 μΜ) prior to the addition of tubulin. This gel is a representation of samples tested in two independent experiments. **e** Fluorescence-based Tubulin Polymerisation Assay in the presence of DCLK1-FL1Δ D511N. Paclitaxel (3 μΜ), a microtubule stabiliser was used as a positive control and nocodazole and tubulin buffer alone were used as negative controls. DCLK1-FL1Δ D511N was incubated with DCLK1-IN-1 (20 and 40 μΜ) or nocodazole (80 μΜ) prior to the addition of tubulin. This curve is a representation of samples tested in duplicates and in two independent experiments. Data points are shown for the first 30 min as tubulin polymerisation reached the steady state. See also Supplementary Fig.10.

Recently, DCLK1-IN-1 was tested against the DCLK1-S isoforms present in certain cancer cells lines and patient derived organoid model of pancreateic cancer^28,37^. However, in pancreatic cancer, over-expression of DCLK1-L isoform has also been reported and is associated with an increase in tumour invasion^40^. Whether DCLK1-IN-1 can also bind to DCLK1-L isoforms and affect DCLK1 tubulin polymerisation function is not known. To begin to unravel this, we first sought to determine if the presence of the N-terminal doublecortin domains impacted DCLK1-IN-1 binding to the DCLK1 kinase domain by thermal shift assay (TSA)^41^. We generated two DCLK1-L (Uniprot O15075-2) constructs: DCLK1-FL1Δ (residues 50-686) and DCLK1-FL2Δ (residues 1-700) (Fig. 5a). Both constructs were generated as WT (phosphorylated), and as a catalytically dead D511N mutant. However, the level of protein recovered from DCLK1-FL2Δ D511N was too little to allow further studies and therefore only tested DCLK1-FL1Δ for its ability to bind DCLK1-IN-1. Similar to DCLK1-KD, the melting temperature of DCLK1-FL1Δ WT and the equivalent D511N mutant increased in the presence of DCLK1-IN-1 compared to the control compound DCLK1-NEG, suggesting binding (Fig. 5b and Supplementary Fig. 8). These data suggest that the DCX domains do not prevent DCLK1-IN-1 from binding to the kinase domain.

### Kinetic mechanism of inhibitor binding explains the lower potency of DCLK1-IN-1

Having established that DCLK1-IN-1 binds to DCLK1-L isoforms (phosphorylated or non-phosphorylated) by TSA, we next wanted directly quantify the affinity of this interaction. We therefore took advantage of the availability of our DCLK1 long construct (DCLK1-FL1Δ; residues 50-686) to carry out a comparative binding analysis of each inhibitor using surface plasmon resonance (SPR), as it would allow simultaneous assessment of both affinity and kinetics. Both the non-phosphorylated (DCLK1-FL1Δ D511N) and phosphorylated wild-type (DCLK1-FL1Δ WT) proteins were immobilised to the sensor chip. We focused our analysis on DCLK1-FL1Δ D511N but similar data were obtained for the WT protein (Supplementary Table 2, Fig. 5c, and Supplementary Fig. 9). The measured binding affinity of DCLK1-IN-1 for DCLK1-FL1Δ D511N (*K*_D_ = 53 nM) (Fig. 5c and Supplementary Table 2) broadly parallels the affinity of DCLK1-IN-1 for DCLK1-KD previously determined by ITC (reported *K*_D_ of 55 and 109 nM in ref. ^37^). In combination with our TSA data (Fig. 5b), this suggests that DCLK1-IN-1 can likely bind with relatively high affinity to either DCLK1-S or DCLK1-L isoforms, in either phosphorylated or non-phosphorylated states. Whilst direct affinity measurements for FMF-03-055-01 and XMD8-85 have not been previously reported, our SPR-determined direct affinity values for FMF-03-055-01 and XMD8-85 binding to DCLK1-FL1Δ D511N (Fig. 5c) showed similar trends to previously reported IC_50_ values for inhibition of DCLK1-KD in a competitive displacement assay^37^, although in our hands XMD8-85 appeared to bind DCLK1 with slightly stronger affinity than that suggested by the reported inhibitory IC_50_. As expected, DCLK1-NEG showed only weak binding to DCLK1 (*K*_D_ > 10 µM). Consistent with earlier observations, we found that by SPR the affinity of DCLK1-IN-1 for DCLK1-FL1Δ D511N (*K*_D_ = 53 nM) was relatively weaker (approximately 4−5 fold) than either FMF-03-055-01 (*K*_D_ = 13 nM) or XMD8-85 (*K*_D_ = 8 nM). This difference is a function of both the on- and off-rates of binding to DCLK1. DCLK1-IN-1 exhibits both a slightly slower on-rate (~2-fold) and a slightly faster off-rate (~2-fold), relative to either FMF-03-055-01 or XMD8-85, for which the kinetics are similar. This difference in kinetics is consistent with our structural data, which suggests that a greater conformational adjustment to the N-lobe of DCLK1 (including opening of the ATP binding site) is required to accommodate DCLK1-IN-1, compared to either FMF-03-055-01 or XMD8-85 (Fig. 4d).

### Binding of DCLK1-IN-1 to the kinase domain does not inhibit DCLK1 MAP function

Having established that DCLK1-IN-1 binds to DCLK1-L isoform with a similar affinity to the DCLK1 kinase domain, we next wanted to show whether DCLK1-IN-1 binding had an impact on DCLK1 MAP function. We and others have previously shown that purified DCLK1 promotes tubulin polymerisation in vitro^21,42^. Our studies have also shown that only a dephosphorylated form of the protein or a catalytically dead mutant, DCLK1-D511N, can polymerise tubulin, implying that DCLK1 kinase activity could play a role in regulating its tubulin polymerisation activity with some phosphorylation sites acting as a regulatory switch^21^. Importantly, we found that most of the human-cancer derived mutations located within the kinase domain would likely adversely impact DCLK1’s kinase domain structural integrity and/or lead to a reduction of its kinase activity^21^. While the biological consequence of these mutations is yet to be fully unravelled, we wanted to understand the impact of the binding of kinase inhibitors on DCLK1 MAP function. We therefore took advantage of the ability of DCLK1-D511N catalytically dead mutant to bind DCLK1-IN-1 and polymerise tubulin to determine if the binding of DCLK1-IN-1 to the kinase domain had an impact on DCLK1 tubulin binding and polymerisation activity. We first performed a phos-tag gel analysis to confirm the difference in phosphorylation between phosphorylated DCLK1-FL1Δ WT and non-phosphorylated DCLK1-FL1Δ D511N (Supplementary Fig. 10a). We utilised two separate assays, microtubule polymerisation and pelleting assays, followed by SDS-PAGE analysis and a fluorescence-based Tubulin Polymerisation Assay (Cytoskeleton)^21^ to test phosphorylated DCLK1-FL1Δ WT and the corresponding catalytically dead mutant D511N. As expected, DCLK1-FL1Δ WT does not polymerise tubulin (Fig. 5d, lanes 1, 2 and Supplementary Fig. 10b) whereas the catalytically dead mutant D511N does, as seen by the increased presence of tubulin (and DCLK1 D511N) in the pelleted fraction (Fig. 5d, lanes 3, 4) and increase in fluorescence due to incorporation of fluorescent reporter in microtubules as polymerisation occurs (Fig. 5e and Supplementary Fig. 10b). This is consistent with our previous observations^21^. Paclitaxel, a microtubule stabiliser which promotes tubulin polymerisation was used as a positive control (Fig. 5e). We next tested tubulin polymerisation in the presence of 20 μM and 40 μM DCLK1-IN-1, concentrations which showed binding to DCLK1 based on our TSA data (Fig. 5b). The presence of DCLK1-IN-1 at 20 μM (Fig. 5d, lanes 5, 7 and Fig. 5e) or 40 μM (Fig. 5d, lanes 8, 9 and Fig. 5e) did not impact DCLK1-FL1Δ D511N’s ability to polymerise tubulin when compared to the control sample without the compound (Fig. 5d, lanes 3, 4 and Fig. 5e). We further demonstrated that the binding of DCLK1-IN-1 to phosphorylated DCLK1-FL1Δ WT did not restore DCLK1-FL1Δ WT tubulin polymerisation activity (Supplementary Fig. 10c). Since some kinase inhibitors are known to stabilise microtubules, we confirmed that DCLK1-IN-1 does not promote tubulin polymerisaton and stabilisation in the absence of DCLK1-FL1Δ D511N, (Fig. 5d, lanes 10, 11 and Fig. 5e). We also confirmed that the addition of 40 μM DCLK1-IN-1 does not cause DCLK1-FL1Δ D511N to aggregate and pellet upon centrifugation (Fig. 5d, lanes 12, 13). As a negative control, we tested tubulin polymerisation in the presence of nocodazole, a well-characterised microtubule destabiliser. As expected, the presence of 40 μM or 80 μM nocodazole inhibits tubulin polymerisation (Fig. 5d, lanes 14, 15 and Fig. 5e). Nocodazole has been suggested to target both kinases and microtubules^43^. We therefore tested whether nocodazole was capable of binding DCLK1 using thermal stability assays (Supplementary Fig. 10d). Nocodazole does not bind DCLK1 at 40 μM and hence the inhibition of tubulin polymerisation observed in the presence of nocodazole can be solely attributed to tubulin destabilisation. These results suggest that the binding of DCLK1-IN-1 to the DCLK1 kinase domain does not affect DCLK1 MAP function. Our studies, therefore collectively indicate that use of DCLK1-IN-1 may be most appropriate in specific cellular contexts where DCLK1 short isoforms are overexpressed, for example, in colon cancer^8,17^, and an inhibition of DCLK1 kinase activity may be required. However, our findings also highlight the need to clearly define the independent contributions of DCLK1 MAP function and kinase function in DCLK1-L isoforms in driving cancer and their interconnectivity to evaluate the broader utility of DCLK1-IN-1.

## Discussion

DCLK1 is widely recognised as a therapeutic target of interest in cancer. DCLK1 deregulation has been reported in various cancers, including gastric, pancreatic, breast, colorectal, and kidney. As outlined, DCLK1 harbours two distinct functions, a kinase and a MAP function and DCLK1 isoforms encode either only one of these two functions or both. There is an emerging appreciation that distinct DCLK1 isoform signatures may exist in cancer development and progression, calling for in-depth studies to rationally design isoform-specific modulators. Recently, the Gray group published DCLK1-IN-1, a selective DCLK1 kinase inhibitor elaborated from a benzopyrimido-diazipinone scaffold, a much-needed tool to dissect the contribution of DCLK1 kinase activity to oncogenesis.

Here, we provide a structural basis for the design of DCLK1-IN-1, including molecular detail to understand how selectivity of DCLK1-IN-1 for DCLK1 is achieved over ERK5 and LRRK2. The structures of DCLK1-KD in complex with the benzopyrimido-diazipinone scaffold analogues XMD8-85 and FMF-03-055-1 show a conventional type I binding mode and in both cases the glycine loop collapses allowing extensive interactions with ring B and ring D contributing to their affinity towards DCLK1. The key positioning of the methyl group in XMD8-85 and the ethyl group in FMF-03-055-1 at position R4 towards the gatekeeper residue, which controls the access to the back pocket within the ATP binding site, along with the difference in the nature of the gatekeeper residue between DCLK1 (Met465) and ERK5 (Leu137) have highlighted how selectivity for DCLK1 over ERK5  can be achieved.

Binding of both XMD8-85 and FMF-03-055-1 stabilised by vdw interactions between ring A and Gly532 that precedes the DFG motif. It is likely that these interactions are contributing to the stabilisation of the activation loop in an active conformation with both the DFG motif and the αC helix adopting a typical active-in conformation stabilised by the canonical salt bridge interaction between β3 strand lysine (Lys419) and the αC helix glutamate (Glu436).

In contrast, in the crystal structure of DCLK1-KD:DCLK1-IN-1, the substitution of the hydrophobic ethyl with an electronegative trifluorethyl group causes an opening of the ATP binding site. This opening causes disruption of the canonical salt bridge interaction between the invariant β3 strand lysine (Lys419) and the αC helix glutamate (Glu436) and an anticlockwise twist of the β-sheets of the N-lobe from the hinge gatekeeper residue. However, despite these notable conformational changes, the DFG-motif retains a ‘DFG-in’ conformation, with Phe534 in this motif stabilising the αC helix in a ‘αC helix-in’ conformation. This unusual binding suggests that DCLK1-IN-1 adopts a conformation akin to a type 1.5 binding mode not previously seen in DCLK1 structures solved to date. These critical features contribute to the formation of a shallow pocket surrounded by the αC helix, the DFG motif and the invariant β3 strand lysine that might be further explored for the design of type 1.5 and type 2 selective DCLK1 inhibitors.

DCLK1-IN-1 shows a clear gain in selectivity towards DCLK1 over ERK5 and the other off-target kinases, albeit a five-fold reduction in DCLK1 affinity compared to FMF-03-055-1. Our SPR data clearly indicate that the reduction in affinity reflects subtly altered binding kinetics, results that are consistent with the observed opening of the binding cleft. Together, this data strongly suggests plasticity of the DCLK1 kinase domain, which can be pushed to adopt an intermediate type 1.5 conformation. An inhibitor with type 1.5 binding mode is likely to be more selective than the typical type I inhibitors, such as XMD8-85 and FMF-03-055-1, as it would capitalise on the structural features distinct to DCLK1.

The selectivity of DCLK1-IN-1 allows the interrogation of the biological kinase functions of DCLK1 isoforms that contain the kinase domain. So far, DCLK1-IN-1 has been shown to be effective in only a subset of patient-derived early stage pancreatic cancer organoid samples that expressed DCLK1-S^37^. The relative expression levels of different DCLK1 isoforms has been shown to vary between the early and late stages in pancreatic ductal adenocarcinoma (PDAC)^44^. It is possible that either a lower DCLK1-S expression, or an altered DCLK1-L expression, in the late stage of pancreatic cancer could explain the lack of DCLK1-IN-1 activity in the remainder of these samples. Our data clearly shows that the binding of DCLK1-IN-1 to non-phosphorylated DCLK1-L does not disrupt the ability of the protein to polymerise tubulin. This is consistent with our previous data demonstrating that DCLK1 kinase activity was necessary to disrupt DCLK1’s tubulin polymerisation function^21^. In addition, our previous structural characterisation of presumed pathological mutations found in DCLK1-L in the context of gastric cancers, also indicated that mutations occurring within the kinase domain would lead to a kinase disfunction^21^. The microtubule-binding affinity of several MAPs (i.e., Doublecortin, Tau, MAP2) and their subcellular localisation have been reported to be tightly regulated by phosphorylation events^5,45^ and in most cases, phosphorylation negatively affects microtubule binding^46^. Our data further confirm a role for DCLK1 kinase activity in controlling DCLK1 tubulin activity and highlight an urgent need for a greater understanding of the expression patterns of DCLK1 isoforms, their differential expression levels during the various stages of cancer, as well as their epigenetic regulation. Regardless, DCLK1-IN-1 represents an invaluable tool to specifically dissect the biological kinase function of the DCLK1-S isoforms and their contribution to tumorigenesis, even as further in-depth biological studies are ongoing. With respect to DCLK1-L isoforms, simultaneous targeting of both the MAP and kinase functions of DCLK1 might be possible through a chemically-induced protein degradation strategy, such as by developing a DCLK1-directed proteolysis-targeting chimera (PROTAC)^47,48^. Importantly, in this case, our inhibitor structures provide a key framework necessary for PROTAC design, in particular to guide linker attachment to a DCLK1 selective kinase-binding moiety, as a handle to recruit DCLK1 for degradation and thereby interrupt both the catalytic and non-catalytic functions of DCLK1.

In conclusion, the DCLK1-KD crystal structures presented here have revealed a remarkable plasticity of the DCLK1 ATP binding pocket, providing a structural framework to guide the design and development of novel isoform-specific modulators as therapeutic agents.

## Material and methods

### DNA constructs

Human DCLK1 (Uniprot O15075-2) constructs for protein expression were cloned into a modified pCOLD vector encoding an N-terminal tobacco etch virus protease cleavage site and an 8XHis tag (Takara). The construct for the catalytically dead mutant was designed using primers containing the mutation, and PCR products  sub cloned into the pCOLD vector. All constructs were verified using Sanger Sequencing (Micromon).

### Recombinant protein expression and purification

Truncated DCLK1 constructs, DCLK1-KD (residues 372-649), DCLK1-Cter (residues 372-686) or DCLK1 FL1Δ (residues 50-686) and DCLK1 FL2Δ (residues 1-700), were expressed overnight at 16 °C for 16−20 h in * Escherichia coli* C41(DE3) as previously described^21^. For crystallisation, proteins were expressed in the presence of lambda phosphatase for homogeneity^21^. DCLK1 proteins were purified by Ni^2+^ affinity chromatography and size exclusion chromatography followed by anion exchange chromatography. Isolated proteins were concentrated to 5 mg/ml and flash frozen in 20 mM Tris pH 7.5, 200 mM NaCl, 5% v/v glycerol, 0.5 mM TCEP.

### Thermal shift stability assay

Thermal shift stability assays were performed as described previously^41^. DCLK1 proteins were diluted in 150 mM NaCl, 20 mM Tris pH 8.0, 1 mM DTT to 2.5 mM final concentration and assayed with the appropriate concentration of inhibitor in a total reaction volume of 25 μL. SYPRO Orange (Molecular Probes) was used as a fluorescence probe and detected at 530 nm. The data shown are representative of two independent experiments.

### Crystallisation, structure determination, and refinement

Crystallographic conditions were modified from Patel et al.^21^. For all the DCLK1-inhibitor complex crystals, purified DCLK1-KD or DCLK1-Cter (5 mg/ml) was pre-incubated with 0.25 mM of compound prior to crystallisation trials. DCLK1-KD was co-crystallised with XMD8-85 and DCLK1-IN-1 at room temperature in 2−4% PEG 400, 1.7−2.3 M ammonium sulphate, 200 mM Hepes pH 6.5−7.25, and 0.5 mM TCEP. DCLK1-KD-Cter (5 mg/ml) was co-crystallised with FMF-03-055-1 at 20 °C in 0.2 M MgCl_2_, 30% PEG 4000, 0.1 M Tris pH 7.75, using the Bio21 C3 Collaborative Crystallisation Centre. Crystals were flash frozen prior to data collection using glycerol (20−25%) as the cryoprotectant. The data were collected at 100 K on the MX2 beamline at a wavelength of 0.9540 at the Australian Synchrotron, Melbourne^49^. The data were processed using iMosflm^50^ and XDS^51,52^, respectively and scaled using AIMLESS from the CCP4 suite^53,54^. The DCLK1 inhibitor structures were solved by the molecular replacement method using PHASER in CCP4^55^, with DCLK1-KD structure (PDB 5JZJ) as a model. The structures were refined using Phenix.refine^56^. Model building was carried out using COOT^57^. The ligand restraints were generated  using the Grade Web Server (Global Phasing Limited). The overall structure were validated using MOLPROBITY^58^. The final model of DCLK1-KD:XMD8-85, DCLK1-Cter:FMF-03-055-1 and DCLK1-KD:DCLK1-IN-1 contained 98.6%, 97.4% and 97.5% residues in favoured regions of the Ramachandran plot, respectively. All molecular graphics representations were created using PyMOL (The PyMOL Molecular Graphics System, Version v1.8.0.3, Schrödinger LLC).

### Surface plasmon resonance

#### Immobilisation

SPR studies were carried out on a Biacore 2000 instrument (Cytivia). Proteins were immobilised at 25 °C using standard amine coupling to a CM5 chip (Cytivia) in immobilisation buffer consisting of 10 mM HEPES pH 7.4, 150 mM NaCl, 1 mM TCEP, 0.005% Tween P20, 1% (v/v) DMSO. To the active flow, cells were coupled either DCLK1-FL1Δ D511N or WT to a final density of approximately 7000 RU, followed by surface deactivation with 1 M ethanolamine. For each protein, coupling was performed at a concentration of 2 µM protein in 10 mM sodium acetate buffer pH 4.0, 1% (v/v) DMSO final, in the presence 10 µM of the moderate affinity compound FMF-03-149-01 (IC_50_ ~400 nM)^28^ to help to stabilise the kinase domain during immobilsiation at low pH. A blank immobilisation was performed on the reference flow cell.

#### Binding studies

Binding experiments were performed at 18 °C in running buffer consisting of 20 mM HEPES, pH 7.4, 150 mM NaCl, 1 mM tris(2-carboxyethyl)phosphine (TCEP), 0.005% (v/v) Tween-P20, and 2% (v/v) DMSO. Compounds (10 mM stocks in 100% DMSO) were diluted in DMSO to 500 µM (DCLK1-NEG) or 25 µM (all other compounds) then in running buffer (without DMSO) to achieve the starting concentration with a final DMSO concentration of 2% (v/v). Samples were then serially titrated in running buffer containing 2% (v/v) DMSO to prepare each final concentration series (seven-point, three-fold serial dilution, 10 µM−13.7 nM for DCLK1-NEG, or 500−0.68 nM for all other compounds). Solutions were injected in multi-cycle format (in duplicate) without regeneration (contact time 80 s, flow rate 75 μL/min, dissociation time 600 s), with an extra wash (50% DMSO) after each injection.

#### Data analysis

Steady-state/kinetic binding data were fitted using the S200 BIAevaluation software (GE Healthcare). Sensorgrams from reference surfaces and blank injections were subtracted from the raw data (double-referencing), followed by solvent correction. Kinetic rate constants (*k*_on_ and *k*_off_ values) were obtained by fitting to 1:1 Langmuir binding model. Reported binding data represent mean ± SEM for three or four independent experiments. The dissociative half-life life (*t*_1/2_) for each inhibitor complex with DCLK1 was calculated from the fitted dissociation rate constant (*k*_off_), according to the equation 1 (*t*_1/2_ = ln(2)/*k*_off._)

### Phos-tag gels

Phos-tag^TM^ pre-cast gel analysis of phosphorylated and non-phosphorylated samples was done according to the manufacturer’s instruction (FUJIFILM Wako Pure Chemical Corporation). DCLK1 WT, DCLK1 WT treated with lamda phosphatase and DCLK1 D511N at 1 μg or 0.5 μg were run on a SuperSep^TM^ Phos-tag^TM^ 12.5% (w/v) SDS-PAGE pre-cast gel. Electrophoresis was carried out at 150 volts for 60 min. The gel was stained using InstantBlue® Coomassie Protein Stain (Expedeon). A conventional 12.5% (w/v) SDS-PAGE analysis with molecular weight markers (Bio-Rad Laboratories) was done in parallel to ensure the proteins for the phos-tag analysis were not degraded.

### Tubulin polymerisation and pelleting assay

Tubulin polymerisation assays were performed in buffer containing 80 mM PIPES at pH 6.9, 2.0 mM MgCl_2_, 0.5 mM EGTA, 2.5 mM β,γ-Methyleneguanosine 5’-triphosphate, and 10 μM tubulin and 5 μM DCLK1 for 1 h at 37 °C. For tubulin polymerisation in the presence of inhibitors, various concentrations of the inhibitors were first incubated with DCLK1 and allowed to stand on ice for 5 min before adding this mix to the tubulin polymerisation reaction. Following the tubulin polymerisation, the reaction mix was pelleted at 20,000 g for 20 min and the supernatant and the pellet fractions were run on a 12% SDS gel. The data shown are representative of two independent experiments.

### Tubulin polymerisation assay

Tubulin polymerisation assay were performed using the Microtubule Polymerisation/Depolymerisation Fluorescence Kit (Cytoskeleton #BK011P) in accordance with the manufacturer’s instructions. DCLK1 (4 μΜ) was added to 50 μL tubulin reaction mix containing Buffer 1 (80 mM PIPES at pH 6.9, 2.0 mM MgCl_2_, 0.5 mM EGTA, 10 μM fluorescent reporter), guanosine triphosphate stock solution at 100 mM, and tubulin (10 mg/ml) stock solution, as described by the manufacturer. Paclitaxel, a control of tubulin polymerisation, was tested at 3 μM, and Νocodazole, a tubulin polymerisation inhibitor, was tested at 80 μΜ. DCLK1-IN-1 was tested at 20 and 40 μΜ with buffer alone as controls. Polymerisation leads to fluorescence enhancement due to incorporation of a fluorescence reporter into polymerising microtubules and was measured by excitation at 355 nm and emission at 460 nm. Readings were taken every 20 s on EnVision Multimode Plate Reader. Each condition was tested in duplicate with two independent batches of protein.

### Reporting summary

Further information on research design is available in the Nature Research Reporting Summary linked to this article.

## Supplementary information

Supplementary information

Reporting summary

## Data Availability

The X-ray structures of DCLK1-KD:XMD8-85 (7KX6), DCLK1-Cter:FMF-03-055-1 (7KX8) and DCLK1-KD:DCLK1-IN-1 (7KXW) have been deposited in the Protein Data Bank. The authors declare that all other data supporting the findings of this study are available within the article and its Supplementary Data files, or from the corresponding authors on request.
