# Supplementary material for: Structural basis for small molecule targeting of Doublecortin Like Kinase 1 with DCLK1-IN-1
Source: Commun Biol. 2021 Sep 20;4:1105. doi: 10.1038/s42003-021-02631-y (PMC8452690; doi:10.1038/s42003-021-02631-y)
Supplement: Supplementary file 1 — Supplementary information [file 42003_2021_2631_MOESM1_ESM.pdf]

Supplementary Information for

Structural basis for small molecule targeting of Doublecortin Like Kinase 1 with DCLK1-IN-1

Onisha Patel[1,2,*], Michael J. Roy[1,2], Ashleigh Kropp[1,2], Joshua M. Hardy[1,2], Weiwen Dai[1,2] and Isabelle S. Lucet[1,2,*]

1. The Walter and Eliza Hall Institute of Medical Research, Parkville, Victoria 3052,Australia
2. Department of Medical Biology, University of Melbourne, Parkville, Victoria, 3052.

*Correspondence: lucet.i@wehi.edu.au, patel.o@wehi.edu.au

This pdf includes:
Supplementary Tables 1 to 2
Supplementary Figures 1 to 10

Supplementary Table 1. List of DCLK1 residues that directly contact DCLK1-IN-1 and of the corresponding residues in ERK5, LRRK2 and PKA. Identical residues are highlighted.

			Corresponding ERK5, LRRK2 and PKA residues		
			**ERK5**	**LRRK2**	**PKA**
**DCLK1**	**Location**	**Interactions**			
V404	β2	side chain	V69	V1893	V57
A417	β3	side chain	A82	A1904	A70
V449	Loop connecting αC to β4	side chain	I117	I1933	V80
M465	Gatekeeper residue	main chain	L137	M1947	M120
E466	Hinge region	main chain	D138	E1948	E121
L467	Hinge region	main chain	L139	L1949	Y122
V468	Hinge region	main chain	M140	A1950	V123
K469	Hinge region	main chain	E141	S1951	A124
G472	Hinge region	main chain	S142	G1953	G125
D475	αD	side chain	Q146	R1957	S130
E515	Loop connecting αE to β6	main chain	S186	H1998	E170
L518	β6	side chain	L189	L2001	L173
G532	Start of the activation loop	main chain	G199	A2016	T183
D533	Start of the activation loop	main chain	D200	D2017	D184

Supplementary Table 2. SPR binding of inhibitors to DCLK1 – summary of fitted values

**Inhibitor**	**Immobilised protein**	**Steady state fitting**			**Kinetic fitting**					
		***K*D (nM)**	**Rmax (RU)**	**N =**	***K*D (nM)**	***k*on (1/Ms) (x 10^5)**	***k*off (1/s)**	**t1/2 (sec)**	**Rmax (RU)**	**N =**
**DCLK1-IN-1**	**DCLK1 FL1Δ D511N**	**70 ± 4**	12 ± 1	4	**53 ± 5**	5.1 ± 0.3	0.027 ± 0.001	26 ± 1	11 ± 1	4
**FMF-03-055-01**	**DCLK1 FL1Δ D511N**	**24 ± 1**	13 ± 1	3	**13 ± 2**	11 ± 4	0.014 ± 0.006	50 ± 20	10 ± 1	3
**XMD8-85**	**DCLK1 FL1Δ D511N**	**14 ± 1**	12 ± 1	3	**8 ± 1**	12.8 ± 0.5	0.010 ± 0.001	67 ± 6	8 ± 1	3
**DCLK1-NEG**	**DCLK1 FL1Δ D511N**	**>10000**		3	**ND**					3
**DCLK1-IN-1**	**DCLK1 FL1Δ WT**	**145 ± 35**	7 ± 1	4	**83 ± 22**	6 ± 2	0.04 ± 0.01	17 ± 4	6 ± 1	4
**FMF-03-055-01**	**DCLK1 FL1Δ WT**	**28 ± 2**	6 ± 1	3	**12 ± 2**	9.6 ± 0.6	0.011 ± 0.001	60 ± 1	4 ± 1	3
**XMD8-85**	**DCLK1 FL1Δ WT**	**12 ± 2**	4 ± 1	3	**7.5 ± 0.3**	22 ± 2	0.016 ± 0.001	44 ± 2	3 ± 1	3
**DCLK1-NEG**	**DCLK1 FL1Δ WT**	**>10000**		3	**ND**					3

Errors are SEM, ND = not determined

K_D, dissociation constant; k_{on}, on-rate; k_{off}, off-rate; $t_{1/2}$, dissociative half-life for the protein/inhibitor complex (calculated from the fitted dissociation rate constant (k_{off}), according the equation $t_{1/2} = \ln 2/k_{off}$); N is the number of independent experiments.

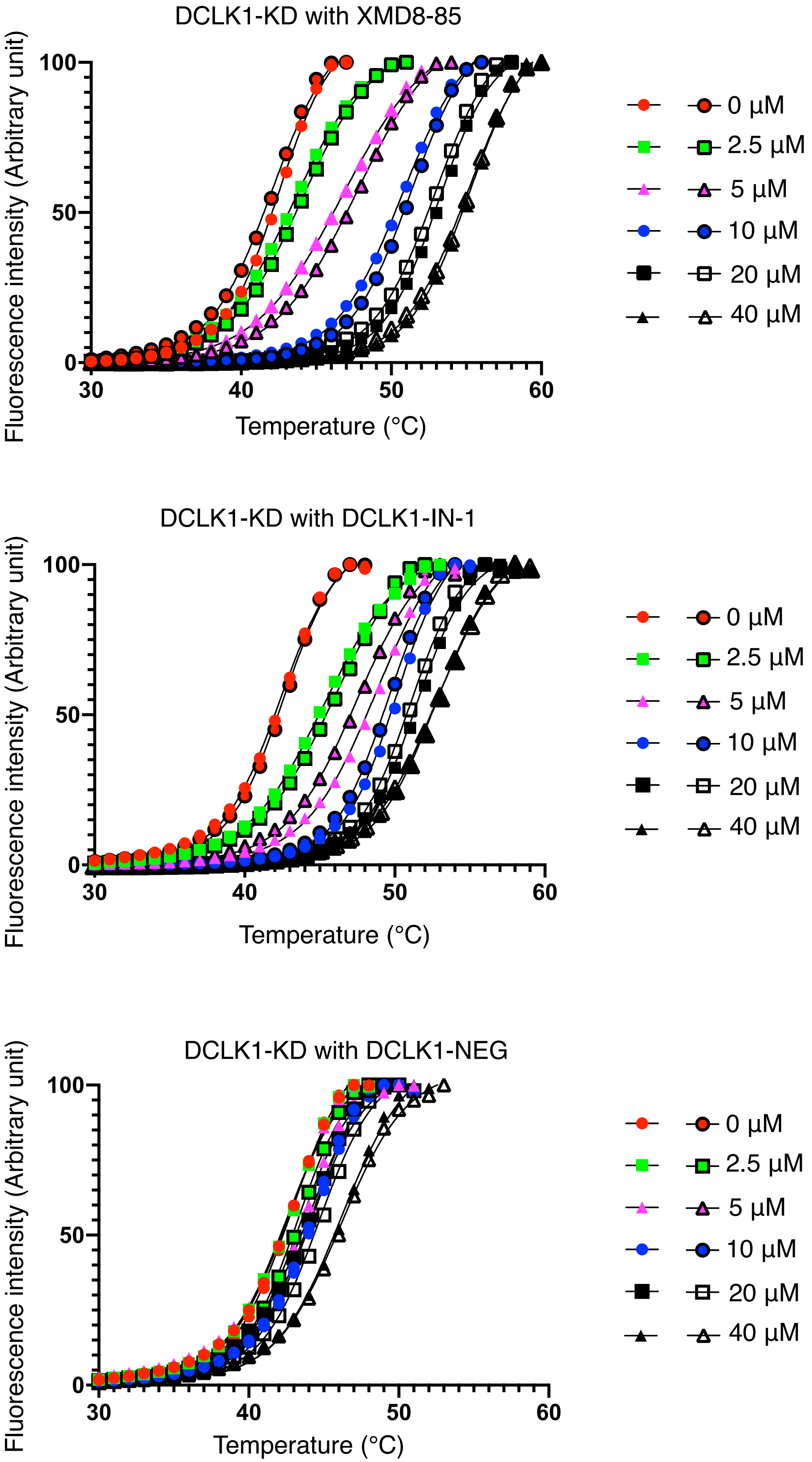

Supplementary Figure 1. Thermal shift assay comparing melting profile of XMD8-85, DCLK1-IN-1 and DCLK1-NEG when bound to DCLK1-KD. Inhibitor concentration tested is as shown. Each curve represents a single inhibitor concentration and each inhibitor concentration is shown in duplicates.

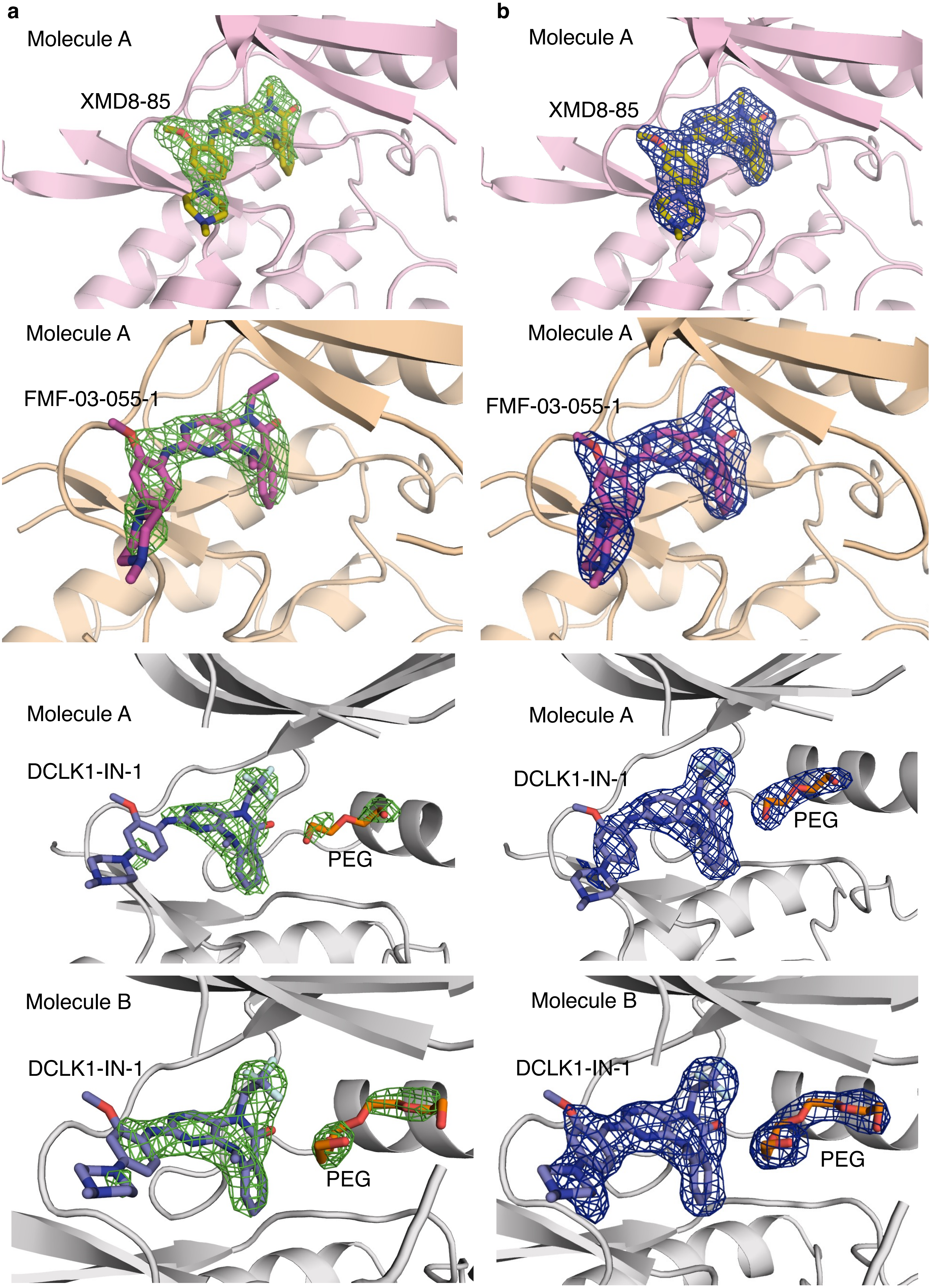

Supplementary Figure 2. **a** Unbiased Fo-Fc density map contoured at 3σ before refinement and model building. **b** 2Fo-Fc map after refinement and model building contoured at 1σ for XMD8-85, FMF-03-055-1 and DCLK1-IN-1 bound to DCLK1. For DCLK1-KD:DCLK1-IN-1, maps for DCLK1-IN-1 and PEG from both molecules (A and B) within the asymmetric unit are shown.

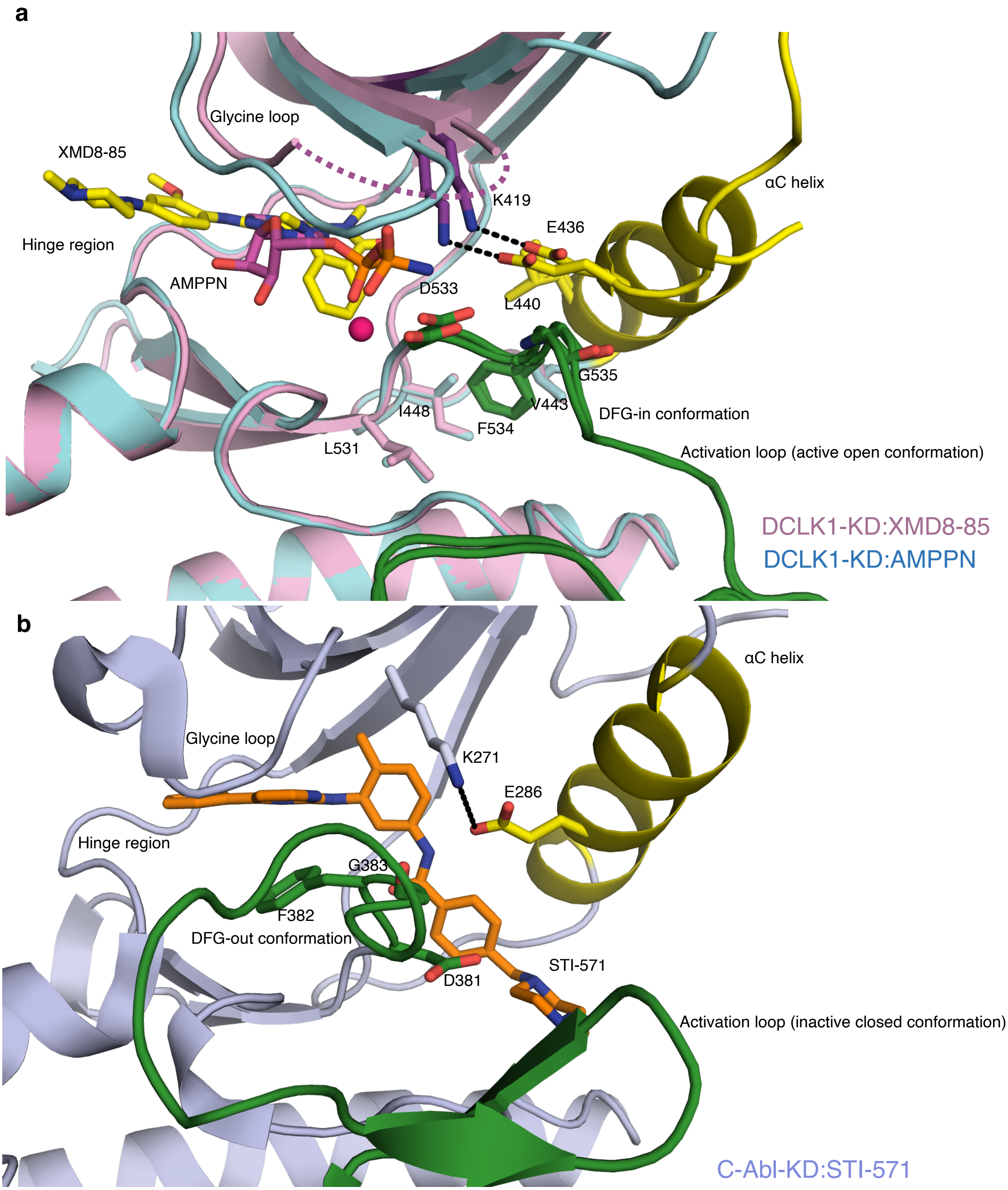

Supplementary Figure 3. **a** Overlay of DCLK1-KD:AMPPN (PDB 5JZJ) and DCLK1-KD: XMD8-85 crystal structures which adapts the kinase active conformation with DFG motif in the DFG-in conformation and activation loop in the active open conformation. **b** C-Abl-KD:STI-571 (PDB 1IEP) with DFG-out conformation and activation loop in the inactive closed conformation.

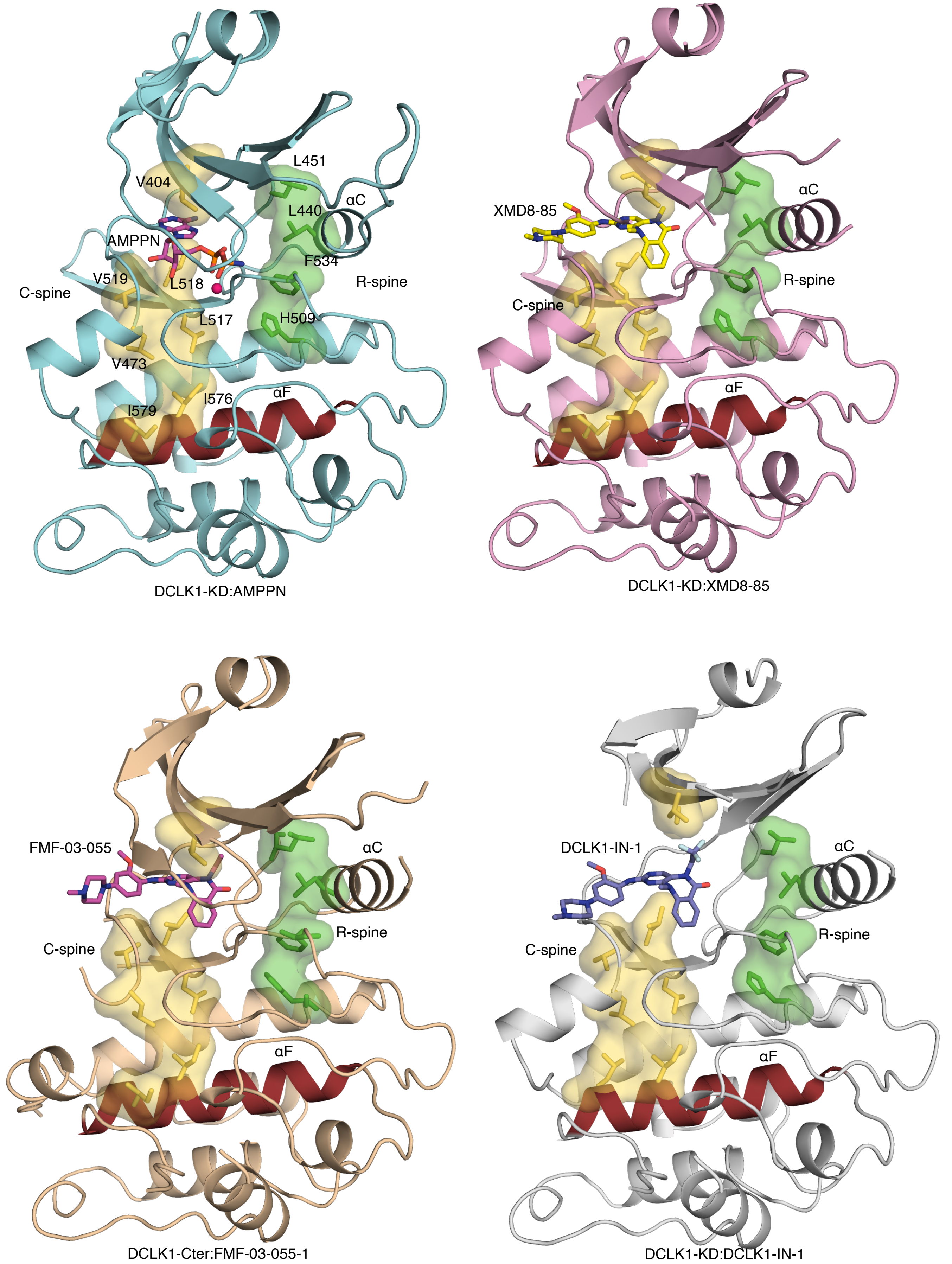

Supplementary Figure 4. Alignment of regulatory (R) spine (residues Val404, Ala417, Leu473, Leu517, Leu518, Val 519, Ile576, Leu580 and catalytic (C) spine (residues Leu440, Leu451, His509 and Phe534) for DCLK1 bound to AMPN (PDB 5JZJ), XMD8-85, FMF-03-055-1 and DCLK1-IN-1. R-spine is shown in green and C-spine is shown in yellow.

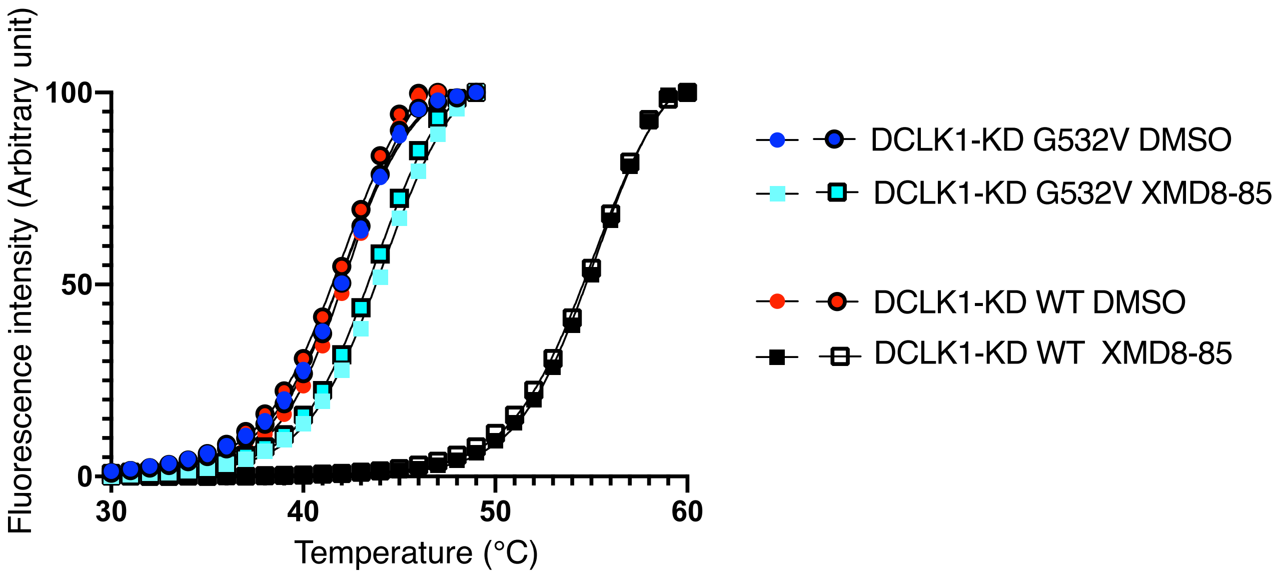

Supplementary Figure 5. Thermal shift assay comparing melting profile of DCLK1-KD WT and DCLK1-KD G532V with XMD8-85 (40 µM) and DMSO control. Curves for each protein is shown in duplicates.

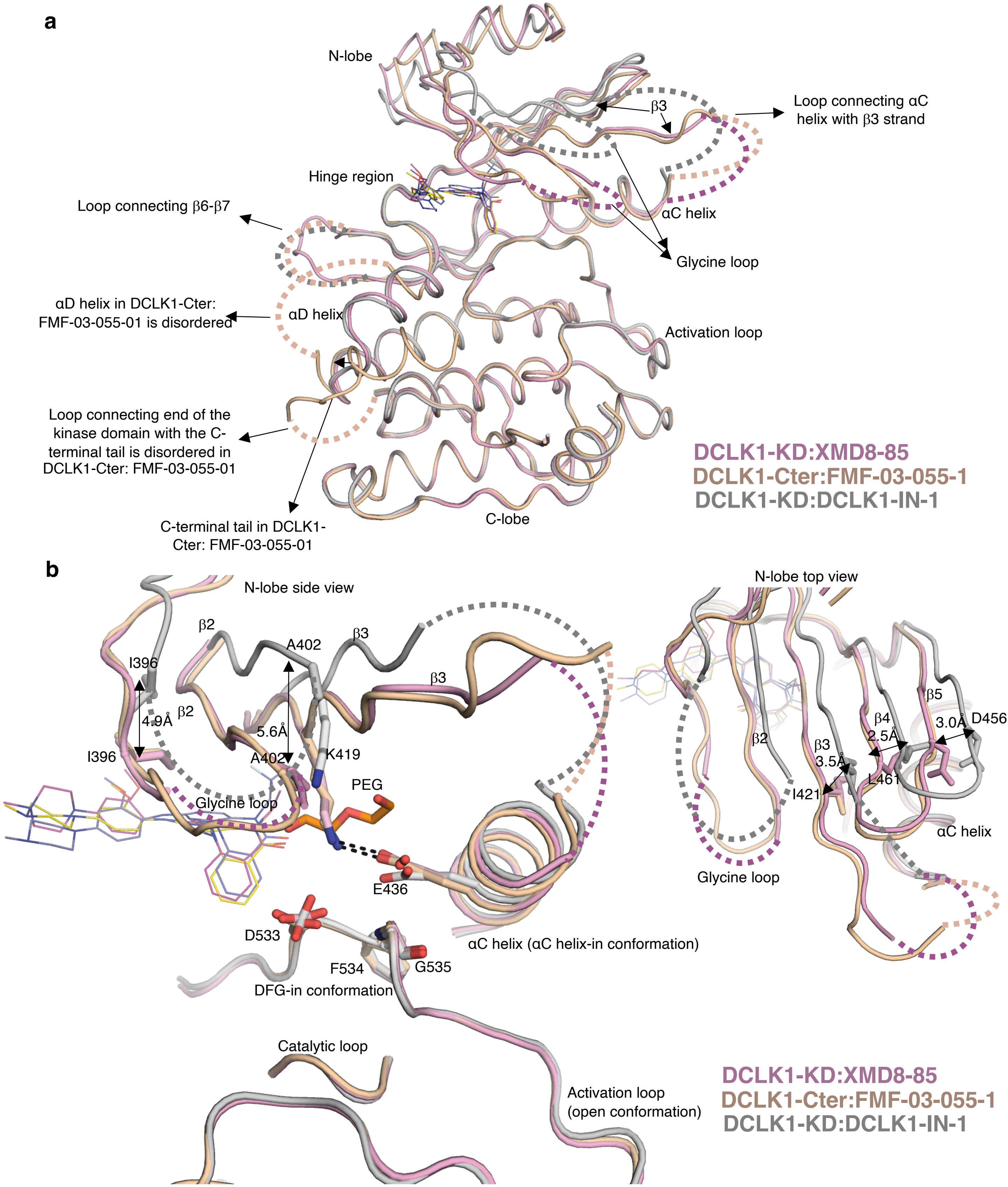

Supplementary Figure 6. **a** Overlay of one molecule of DCLK1-KD: XMD8-85, DCLK1-Cter:FMF-03-055-1 and DCLK1-KD:DCLK1-IN-1. Disordered loops are highlighted in dashed lines. The root mean square deviation after superposition of DCLK1-KD:DCLK1-IN-1 with DCLK1-KD:XMD8-85 is 0.261Å over 190 Cα atoms and with DCLK1-Cter:FMF-03-055-1 is 0.316Å over 175 Cα atoms. **b** Left, the opening of the ATP binding pocket in DCLK1-KD:DCLK1-IN-1 results in an upward shift of up to 5Å near the glycine loop highlighted at the Cα atom of Ile396 at the start of the glycine loop and Ala402 at the end of the glycine loop. The position of the αC helix, the DFG motif, the activation loop and the catalytic loop does not change in the three structures. Right, the N-lobe in DCLK1-KD:DCLK1-IN-1 undergoes a twist that shifts the position of β strands between 2.5-3.5Å compared to DCLK1-KD:XMD8-85 and DCLK1-Cter:FMF-03-055-1.

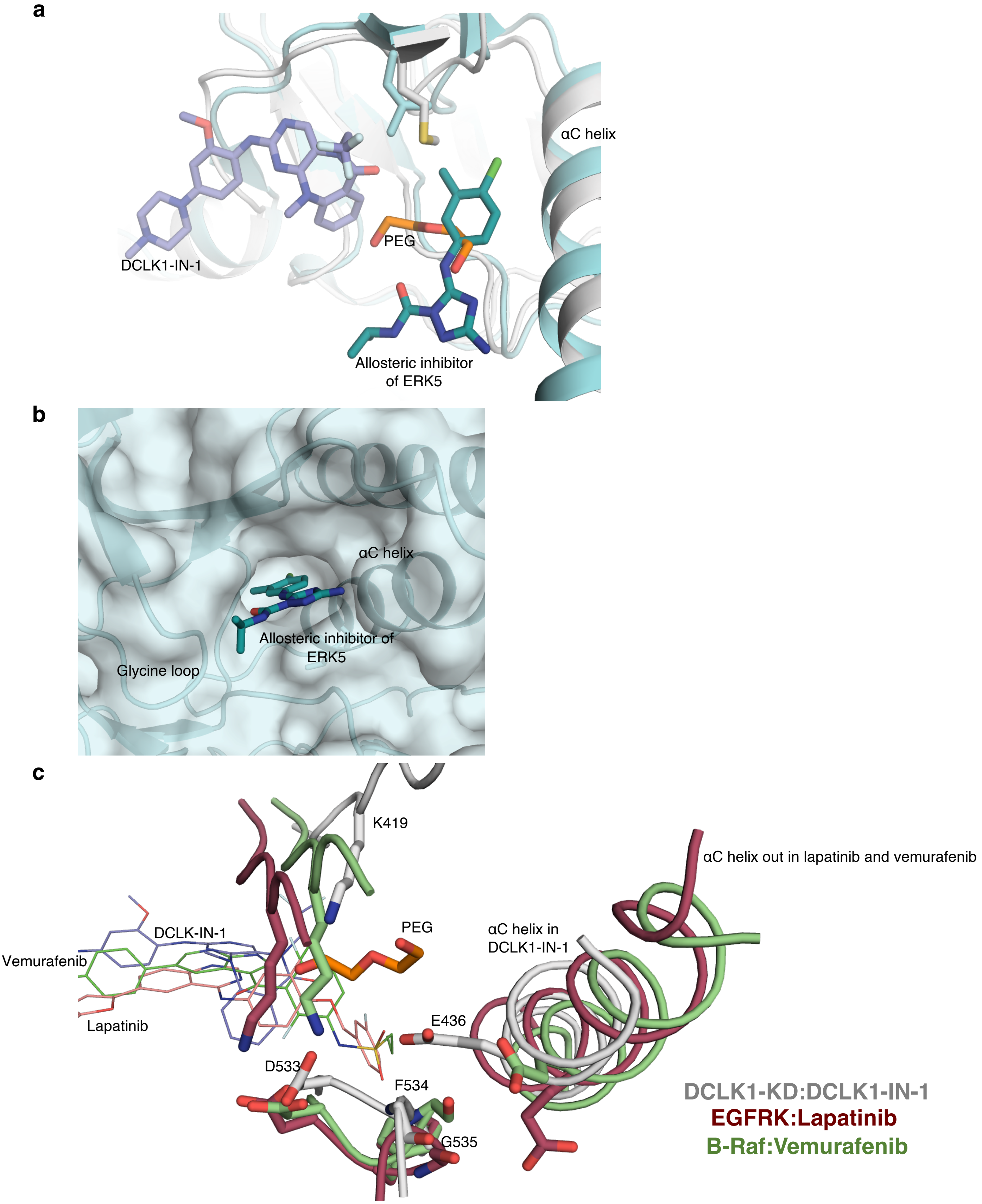

Supplementary Figure 7. **a** Overlay of DCLK1-KD:DCLK1-IN-1 with ERK5 structure bound to an allosteric inhibitor (PDB 4ZSJ). The position of the PEG molecule in DCLK1-KD:DCLK1-IN-1 aligns with the position of the allosteric inhibitor in ERK5. **b** The crystal structure of ERK5 with an allosteric inhibitor (PDB 4ZSJ) showing the allosteric pocket stabilised in between the glycine loop and the αC helix. **c** Overlay of DCLK1-KD:DCLK1-IN-1 with type 1.5 inhibitors, EGFRK:Lapatinib (PDB 1XKK) and B-Raf:Vemurafenib (PDB 3OG7) to highlight the disruption of canonical salt bridge between glutamate and lysine. EGFRK:Lapatinib and B-Raf:Vemurafenib has the αC helix out conformation while DCLK1-KD:DCLK1-IN-1 has the αC helix in conformation.

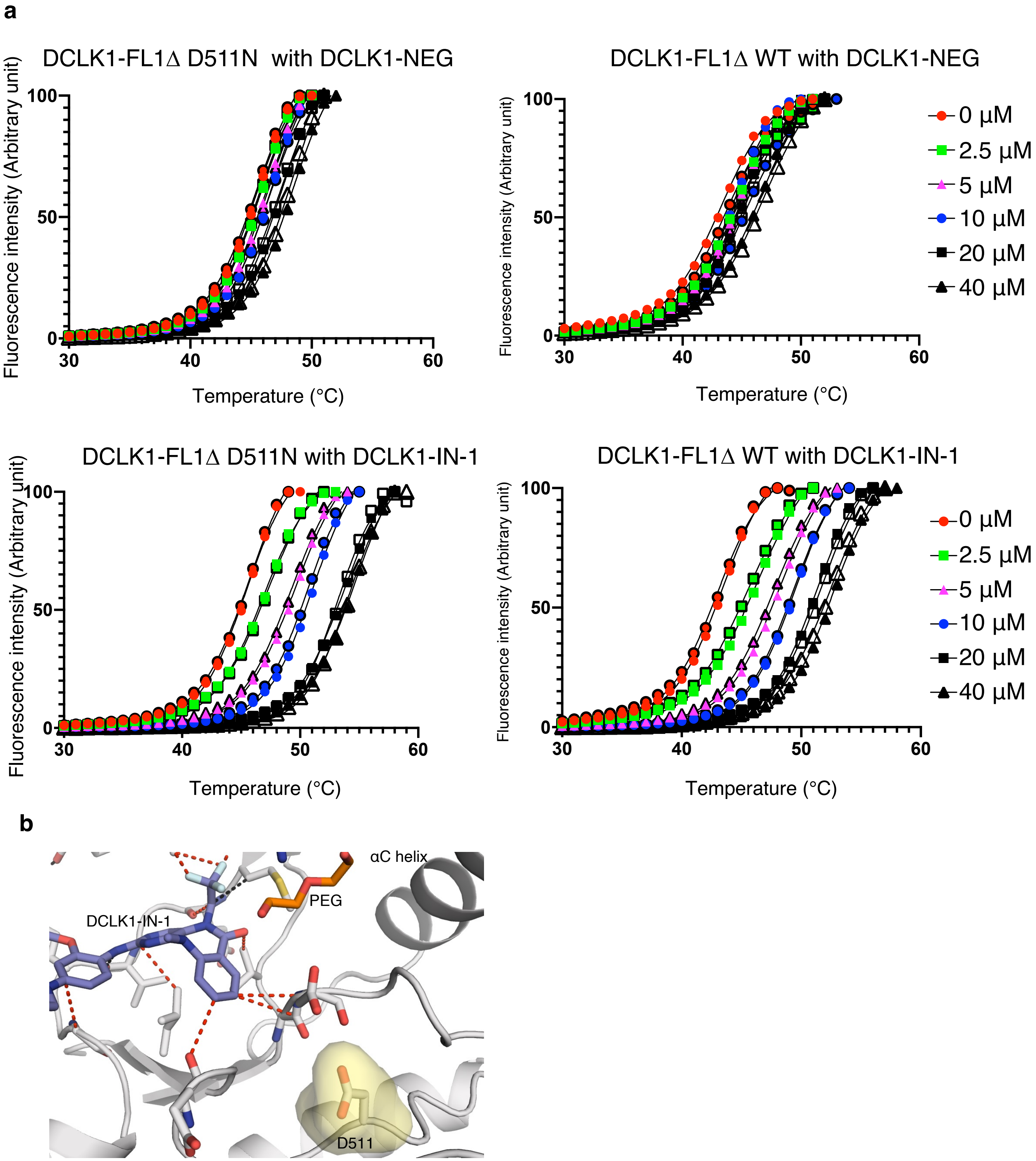

Supplementary Figure 8. **a** Thermal shift assay comparing melting profile of DCLK1-IN-1 and DCLK1-NEG with DCLK1-FL1Δ WT and DCLK1-FL1Δ D511N. Each curve represents a single concentration and each concentration is shown in duplicates. **b** Location of D511N mutant (yellow) in the catalytic loop to show inhibitor binding with this mutant will be unaffected.

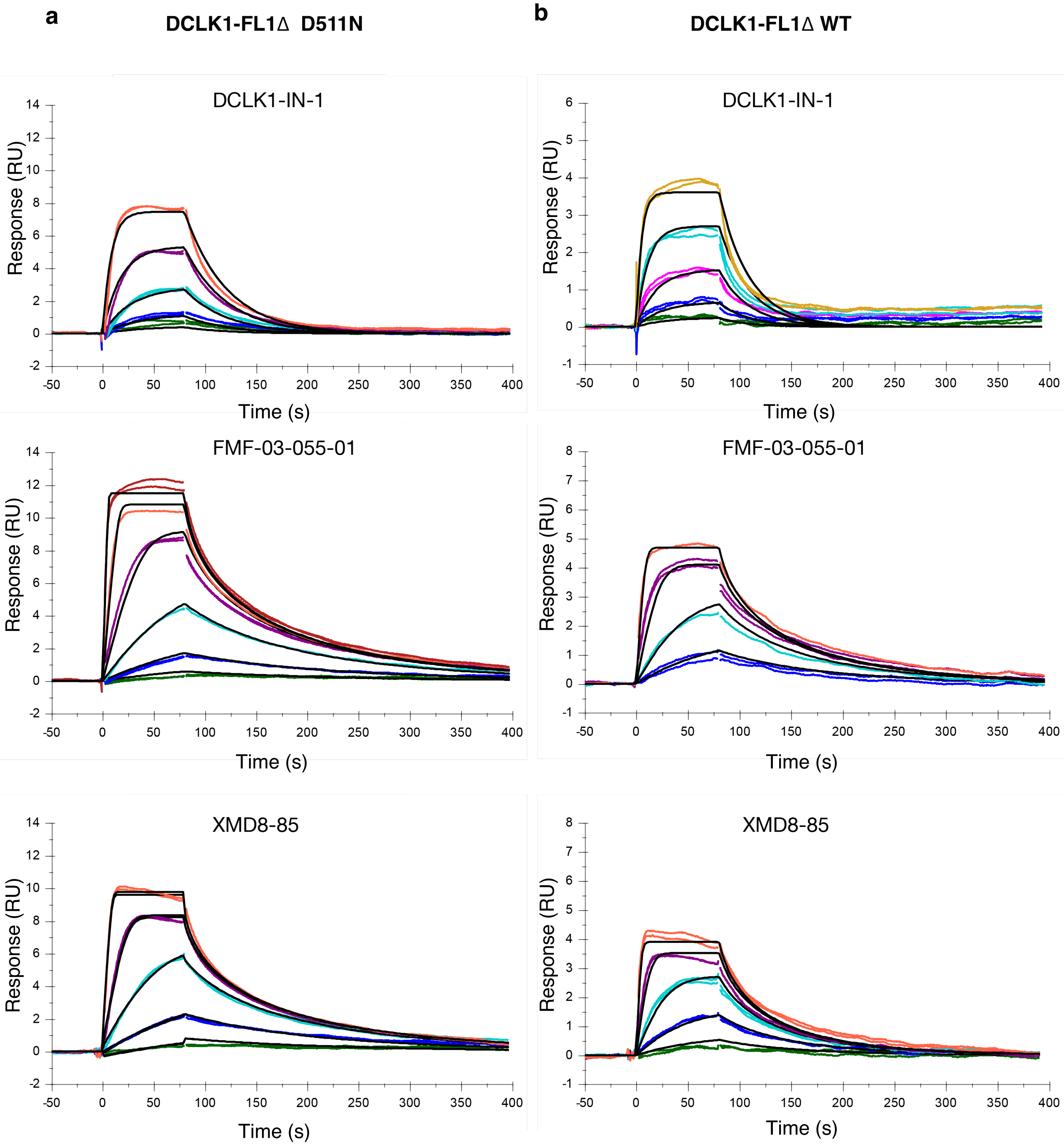

Supplementary Figure 9. SPR kinetic fitting. **a** Representative fitted SPR sensorgrams for DCLK1-IN-1, FMF-03-055-1 or XMD8-85 binding to immobilised DCLK1-FL1Δ D511N. **b** Representative fitted SPR sensorgrams for DCLK1-FL1Δ WT. Black lines represent kinetic fitting using a 1:1 binding model. Data represents an average of either four (DCLK1-IN-1) or three (FMF-03-055-01 and XMD8-85) independent experiments. Mean fitted values are listed in Supplementary Table 2.

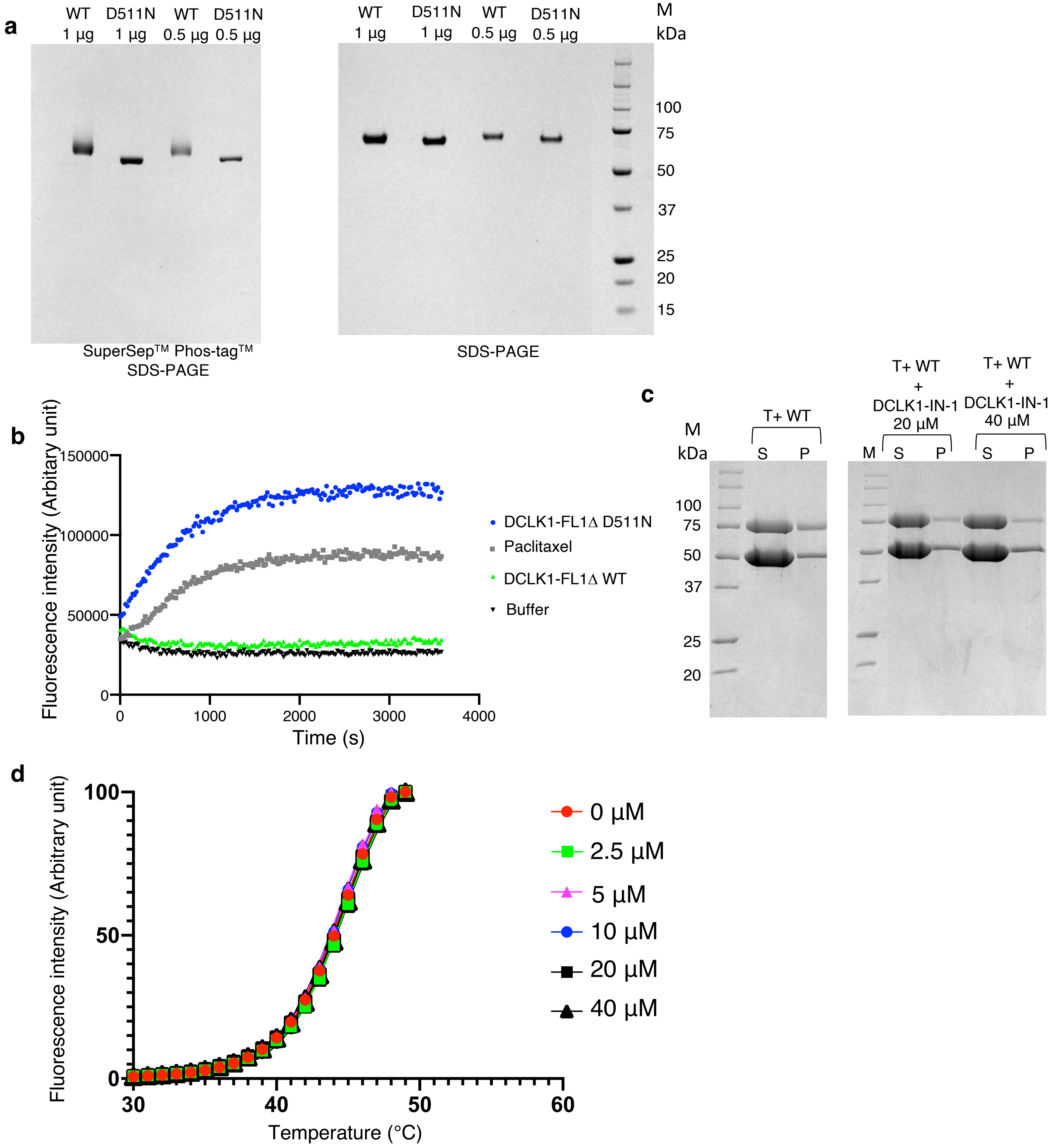

Supplementary Figure 10. **a** SuperSep™ Phos-tag™ 12.5% SDS-PAGE gel (left) and SDS-PAGE analysis (right) of DCLK1-FL1Δ WT and DCLK1-FL1Δ D511N. 1 µg and 0.5 µg samples were loaded on the gel. An SDS-PAGE gel with molecular weight markers was run in parallel to make sure the proteins for the phos-tag analysis were not degraded. **b** Tubulin polymerisation assay. Tubulin was incubated alone (control buffer), with paclitaxel (3 µM), or with DCLK1-FL1Δ WT (4 µM) and DCLK1-FL1Δ D511N (4 µM). This curve is a representation of samples tested in duplicates and in two independent experiments. **c** SDS-PAGE gel analysis of pellet (P) and supernatant (S) fractions following tubulin polymerisation in the presence of DCLK1-FL1Δ WT incubated with DCLK1-IN-1 at 20 and 40 µM. DCLK1-FL1Δ WT (WT) was incubated with DCLK1-IN-1 before adding tubulin (T). This gel is a representation of samples tested in two independent experiments. **d** Thermal shift assay to show that DCLK1-FL1Δ D511N does not bind tubulin destabilisation drug, nocodazole (40 µM). This curve is a representation of samples tested in duplicates.
